# β-Alkenylation of Saturated *N*-Heterocycles via a C(sp^3^)–O Bond Wittig-like Olefination

**DOI:** 10.1021/acs.joc.3c02466

**Published:** 2024-01-12

**Authors:** Ángel A. Nolasco-Hernández, Leticia Quintero, Silvano Cruz-Gregorio, Fernando Sartillo-Piscil

**Affiliations:** Centro de Investigación de la Facultad de Ciencias Químicas, Benemérita Universidad Autónoma de Puebla (BUAP), 14 Sur Esq. San Claudio, Col. San Manuel, 72570 Puebla, México

## Abstract

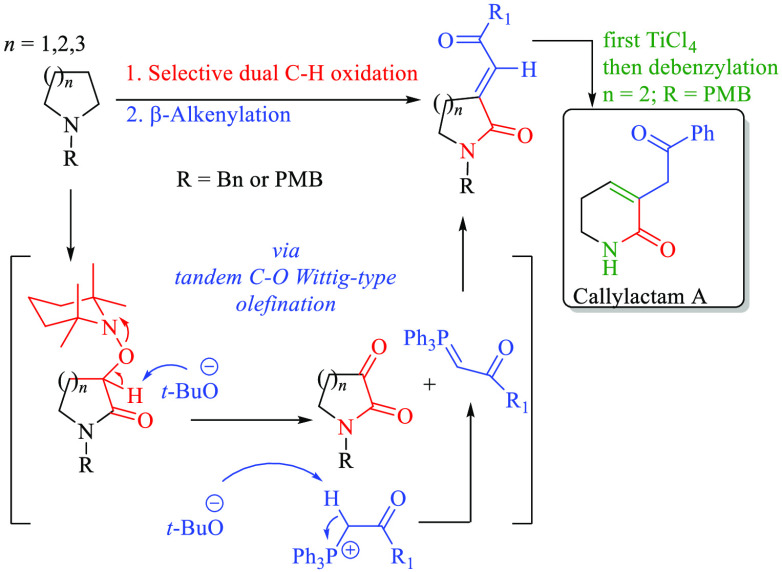

Although the C–H_α_ functionalization of *N*-heterocycles is, in fact, an easy chemical transformation, the C–H_β_ functionalization is, on the contrary, a quite difficult chemical process. Here, we present a two-step protocol that allows the ready conversion of pyrrolidines, piperidines, and an azepane into their corresponding 3-*exo*-alkenyl lactams via the transient formation of 3-alkoxyamino lactams followed by a Wittig-like C(sp^3^)–O bond olefination with stabilized ylides from phosphonium salts mediated by *t*-BuOK. Additionally, as a proof of the synthetic effectiveness of this novel methodology, the first synthesis of the natural product callylactam A was achieved through a TiCl_4_-catalyzed double bond isomerization of a 3-*exo*-alkenyl 2-piperidone to its *endo-*isomer.

## Introduction

At the present time, the C–H functionalization of unreactive bonds is recognized as one of the most important chemical operations within the state-of-the-art of organic synthesis.^1^ Nevertheless, this transformation is even less popular than the traditional refunctionalization process. As such, it has not been officially incorporated into the paradigmatic use of the retrosynthetic analysis, neither as a functional group interconversion nor as a bond disconnection.^2^

**Scheme 1:**
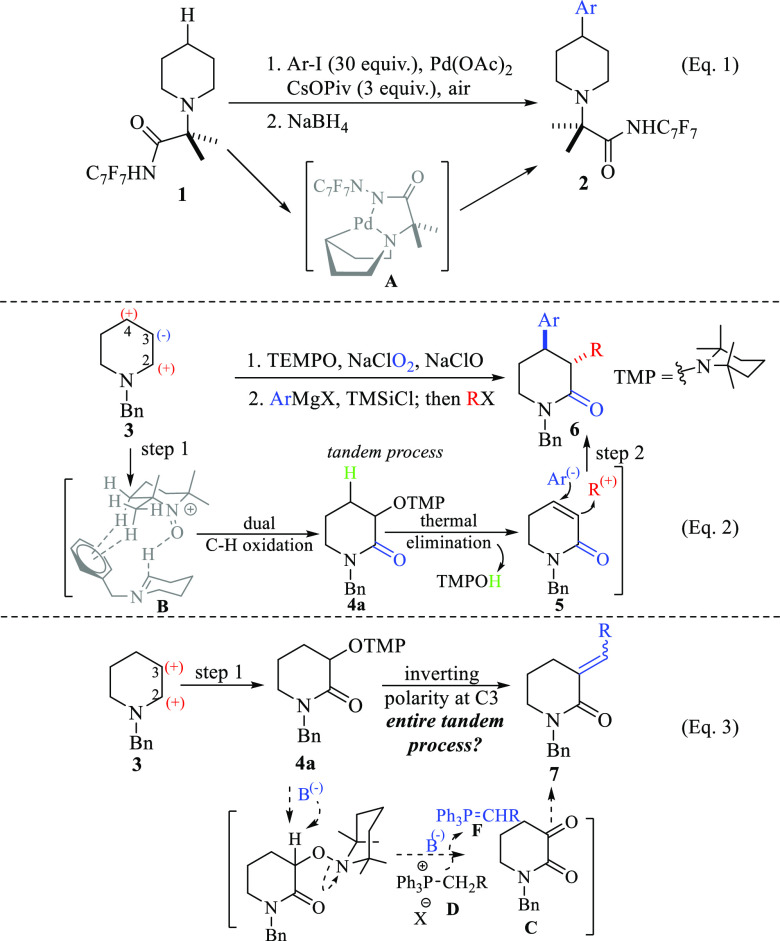
Transition-Metal-Catalyzed C–H Arylation of Piperidines (Eq 1); Transition-Metal-Free (TMF) C–H Functionalization of Piperidines (Eq 2); Proposed TMF β-Alkenylation of Piperidines (Eq 3)

Moreover, this synthetic strategy has been effectively utilized, not only in lineal synthesis but also in the late-stage diversification of relevant natural products.^3^ For instance, Sanford and co-workers^4^ envisaged the remote CH_2_ group of cyclic amines to connect aryl groups through a palladium-catalyzed C–H arylation reaction (**1** → **2**) offering thus a rapid access to relevant pharmaceutical alkaloids (Scheme 1; eq 1).^4^ In contrast, an alternative transition-metal-free (TMF) version is also available, not only for mono but also for a dual C–H functionalization process. Since the latter protocol visualizes the C4 and C3 positions as dipolar synthons (**3**), nucleophiles and electrophiles were incorporated in a tandem fashion (**5** → **6**) (Scheme 1, eq 2).^5^ Unlike the transition-metal catalyzed reactions, in which the directing group is majorly responsible for the reaction success (e.g., **A**), in the TMF version, the benzyl group of piperidine **3** has been proposed to act as a pseudodirecting group (**B**)^6^ for the selective dual C–H oxidation mediated by oxoammonium cation (TEMPO^+^) to the transitory intermediate alkoxyamino lactam **4a,** which is crucial for the third C–H functionalization (**5**), and thus to access to 3,4-disubstituted-2-piperidones **6** in only two-steps from simple piperidines.

## Results and Discussions

On these bases, it was envisioned to invert the polarity at the C3 position of piperidine **3** (and related *N*-heterocycles) to incorporate a suitable carbon-based nucleophile.^7^ Accordingly, it was hypothesized that with the action of a base, it could simultaneously mediate the deamination process of **4a** to **C** and the formation of phosphorus ylide **F** from phosphonium salt **D**, to carry out olefination at C3 of **C**. Since this entire chemical transformation is planned to be performed under a tandem fashion, we propose that this chemical transformation could be termed as a Wittig-like olefination of a C(sp^3^)–O bond from **4a**.

Execution of the proposed idea was performed with *N*-benzyl piperidine **3**. The first step, which involves the selective and dual C–H oxidation of **3** to **4a** can be successfully achieved by following any of the two reported protocols,^8,9^ one in which the oxoammonium cation is formed in situ by reacting TEMPO with NaClO_2_ and NaOCl (Method A),^8^ or by using TEMPO^+^ cation freshly prepared plus NaClO_2_ (Method B).^9^ Subsequently, the phosphonium salt [Ph_3_P^+^CH_2_CO_2_Me]Br^–^, which produces the corresponding stabilized P-ylide under base conditions, was selected for testing the title reaction.

Accordingly, when piperidine **3** was transformed into transient alkoxyamino lactam **4a** and then subjected to react with sodium methoxide in methanol, from room temperature (rt) to 60 °C for 18 h, the expected alkenylated product **8** was not obtained (Table 1, entry 1). The result was practically the same with NaOH in aqueous methanol (entry 2). However, traces of the product with concomitant decomposition of the starting material were obtained by using *n*-BuLi in tetrahydrofuran (THF) at low temperatures (entry 3). A modest mixture of *E*-**8**/*Z*-**8** (∼2/1) was obtained with *sec*-BuLi at −40 °C (entry 4), and no changes were observed by increasing the equivalents of phosphonium salt (entry 5). Other bases like lithium bis(trimethylsilyl)amide (LiHDMS) (entry 6) and lithium diisopropylamide (LDA) (entry 7) did not produce the expected product, and alkoxyamino lactam **4a** remained unchanged even with stirring for up to 20 h. Fortunately, we were pleased to find that substrate **4a** reacted with 7 equivalents of potassium *t*-butoxide (*t*-BuOK) in solution and 3 equivalents of phosphonium salt giving the expected olefinated product *E*-**8** in good 78% yield and high *E*-selectivity (entry 8). Attempts to improve the chemical yield by increasing the temperature (entry 9) or the amount of the phosphonium salt (entry 10) were unproductive. It was also explored the alkenylation by preparing the stabilized ylide outside the reaction flask and then added to the reaction mixture giving practically the same yield, albeit it took longer time to consume the starting material (entry 11). It is important to note that the alkenylation reaction at the gram scale (using 1.8 g, 5.2 mmol of **4a**) with [Ph_3_P^+^CH_2_CO_2_Me]Br^–^ salt gave the same chemical yield obtained in entry **8** (see Supporting Information for experimental details).

**Table 1:**
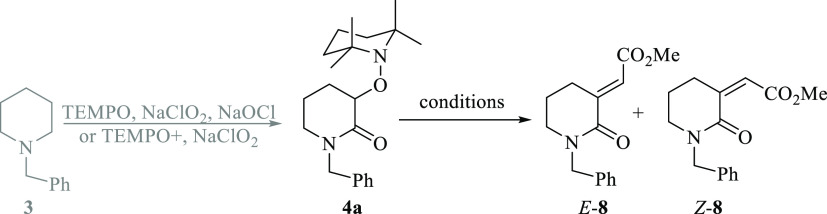
Optimization of the C-3 Alkenylation of Piperidine **3** via Wittig-like Olefination of a C(sp^3^)–O Bond of Transient Alkoxyamino Lactam **4a**^a^

entry	base (equiv)	phosphonium salt (equiv)	solvent	temperature (°C)	time (h)	yield (%)^b^
1	MeO^–^Na^+^ (6)	[Ph_3_P^+^CH_2_CO_2_Me]Br^–^ (3)	MeOH	rt to 60	18	c
2	NaOH (10)	[Ph_3_P^+^CH_2_CO_2_Me]Br^–^ (3)	MeOH/H_2_O	rt to 60	18	c
3	*n*-BuLi (3)	[Ph_3_P^+^CH_2_CO_2_Me]Br^–^ (3)	THF	–40	1	traces
4	*sec*-BuLi (3)	[Ph_3_P^+^CH_2_CO_2_Me]Br^–^ (3)	THF	–40	1	∼8; *E/Z* = 2/1
5	*sec*-BuLi (6)	[Ph_3_P^+^CH_2_CO_2_Me]Br^–^ (5)	THF	–40 to rt	3	∼8; *E/Z* = 2/1
6	LiHMDS (6)	[Ph_3_P^+^CH_2_CO_2_Me]Br^–^ (3)	THF	0 to rt	14	c
7	LDA (6)	[Ph_3_P^+^CH_2_CO_2_Me]Br^–^ (3)	THF	0 to rt	20	c
**8**	*t***-BuOK (7)**	**[Ph**_**3**_**P**^**+**^**CH**_**2**_**CO**_**2**_**Me]Br**^**–**^ **(3)**	*t***-BuOH**	**0 to rt**	**8**	**78; only***E*
9	*t*-BuOK (6)	[Ph_3_P^+^CH_2_CO_2_Me]Br^–^ (3)	*t*-BuOH	rt to 45	6	36; only *E*
10	*t*-BuOK (6)	[Ph_3_P^+^CH_2_CO_2_Me]Br^–^ (5)	*t*-BuOH	rt to 45	6	32; only *E*
11^d^	*t*-BuOK (4)	[Ph_3_P^+^CH_2_CO_2_Me]Br^–^ (3)	*t*-BuOH	0 to rt	13	72; only *E*

a Reactions were performed using 0.05 M solution of 3-alkoxyamino lactam in anhydrous *t*-BuOH at room temperature (unless otherwise stated).

b Yields are reported after silica gel chromatography.

c Starting material remains unchanged.

d The corresponding stabilized ylide was preformed from its phosphonium salt and it was added to the reaction mixture.

Having developed an efficient two-step protocol for the C-3 *exo*-alkenylation (olefination) of piperidine **3** (Table 1 entry 8), we explored the scope of this novel protocol to a series of selected substrates of which some of them are intended to be precursors for projected total synthesis of relevant alkaloids. Accordingly, benzylated pyrrolidine fully saturated **9** was transformed into olefin products **10** and **11** in moderated yields by using phosphonim salts [Ph_3_P^+^CH_2_CO_2_Me]Br^–^ and [Ph_3_P^+^CH_2_COMe]Br^–^, respectively (Table 2, entries 1 and 2).

**Table 2:**
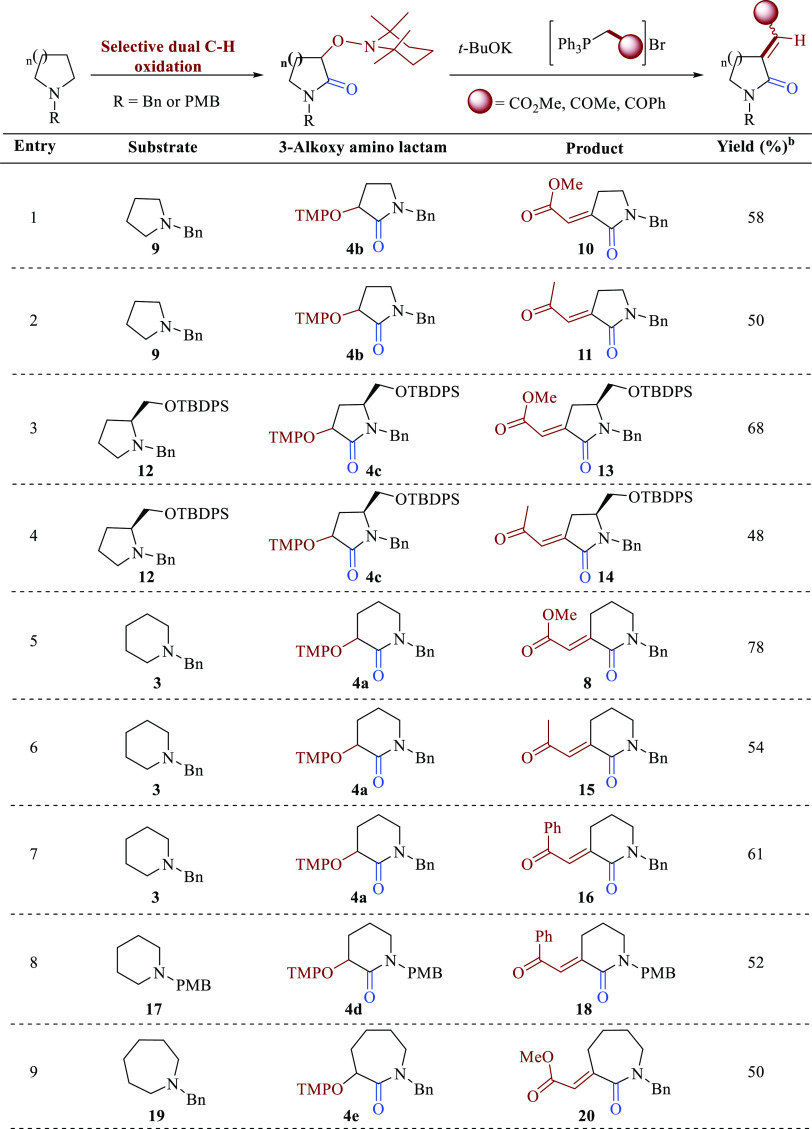
Developing of TMF C-3 Alkenylation of *N*-Benzyl Heterocycles

a Reactions were performed using 0.05 M solution of 3-alkoxyamino lactam in anhydrous *t*-BuOH at room temperature (unless otherwise stated).

b Yields are reported after silica gel chromatography.

Moving to chiral pyrrolidines derived from *L*-proline **12** resulted in the formation of potential alkaloid precursors **13** and **14** in good and moderated chemical yields (entries 3 and 4, respectively). Unlike benzyl piperidine **3**, where the alkenylation with phosphonium salt [Ph_3_P^+^CH_2_CO_2_Me]Br^–^ gave **8** in good chemical yield (entry 5), related phosphonium salt [Ph_3_P^+^CH_2_COMe]Br^–^ gave moderated yield of **15** (entry 6). Similar chemical yields were obtained for piperidines **3** and **17** with [Ph_3_P^+^CH_2_COPh]Br^–^ to obtain alkenylated products **16** and **18**, respectively (entries 7 and 8). It was also explored this TMF alkenylation with benzyl azepane **19** and [Ph_3_P^+^CH_2_CO_2_Me]Br^–^, and the respective C–H olefinated product **20** was obtained in moderate chemical yield (entry 9).

**Scheme 2:**
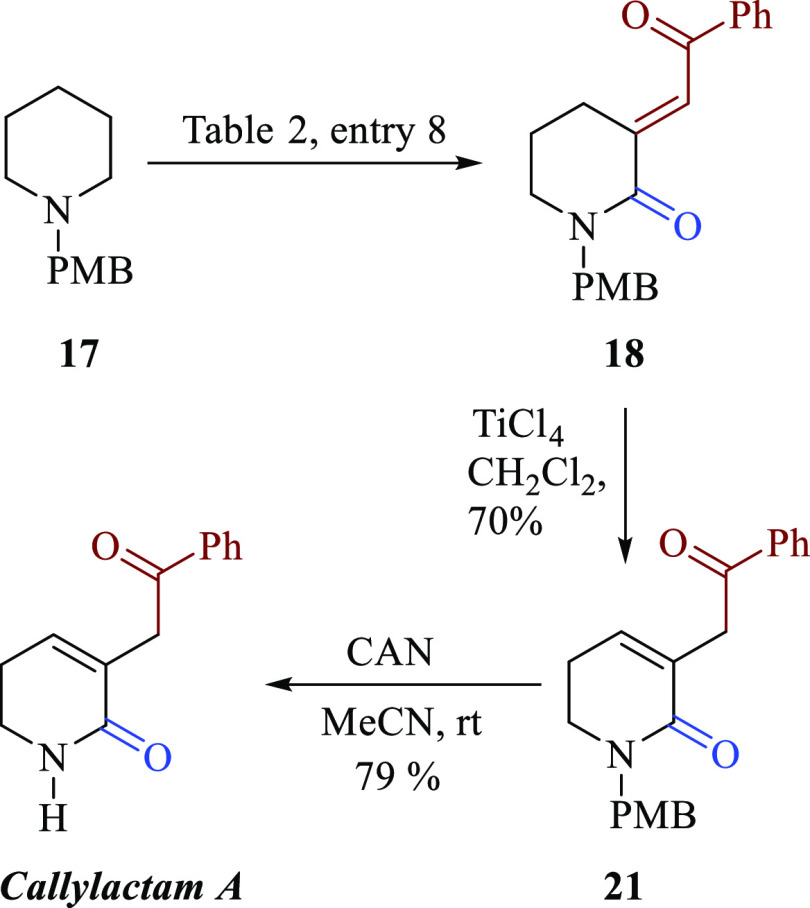
Application of the TMF C-3 Alkenylation of *N*-benzyl Piperidine **17** to the Total Synthesis of Callylactam A

Finally, as a proof of the synthetic potential of this novel selective and dual C–H functionalization of saturated N-heterocycles, the first total synthesis of the natural product isolated from the marine sponge of the genious *Callyspongia spp* (callylactam A)^10^ was obtained in only three steps from piperidine **17** (Scheme 2).

To achieve this aim, it was necessary to isomerize the *exo*-double bond in **18** to the *endo*-double bond (**21**). Accordingly, a Lewis acid double bond isomerization was explored.^11^ Fortunately, with the use of equimolar amount of TiCl_4_ in CH_2_Cl_2_ at rt, the expected product **21** was obtained in 70% yield, Finally, with the removal of *p*-methoxy benzyl group (PMB) with ceric ammonium nitrate (CAN), the first total synthesis of callylactam A was accomplished.

## Conclusions

In summary, we have developed, under TMF conditions, an efficient and accessible synthetic methodology for the C–H alkenylation of saturated *N*-heterocycles to (*E*)-3-*exo*-alkenyl lactams. Additionally, with the application of a Lewis acid-catalyzed double bond isomerization of a 3-e*xo*-double bond into its *endo*-isomer, we achieved the first total synthesis of callylactam A in only 3 steps from a saturated piperidine. Accordingly, this opens the possibility of further functionalization of simple *N*-heterocycles into a wide variety of alkaloid precursors.

## Experimental Part

Commercially available reagents were used without further purification. Unless otherwise noted, reactions were carried out under an inert argon atmosphere with dry solvents under anhydrous conditions. Solvents were used as technical grade and freshly distilled prior to use. Reactions were monitored by thin-layer chromatography (TLC), which was monitored by ultraviolet (UV) light. Purification of products was performed by column chromatography (CC) using silica gel (230–400 mesh) with solvents indicated in the text. Melting points were not corrected and were carried out on a Fisher-Scientific 12–144 melting point apparatus. Nuclear magnetic resonance (NMR) spectra were recorded on a Bruker-500 (500 MHz) using as reference: TMS for ^1^H (0.0 ppm), the residual solvent peak of CDCl_3_ for ^13^C (77.16 ppm); chemical shifts (δ) are stated in parts per million (ppm) and Hz for the coupling constants (*J*). The following abbreviations (or combinations thereof) were used to explain the multiplicities: s = singlet, d = doublet, t = triplet, q = quartet, quint = quintet, m = multiplet, and br = broadened.

### General Procedure for the Preparation of Benzyl Amines **9**, **3**, and **19**

To a stirred suspension of K_2_CO_3_ (52.4 mmol) in dry CH_3_CN (100 mL) at rt was added the respective cyclic amine (pyrrolidine, piperidine, or azepane) (40 mmol). The reaction mixture was stirred for 20 min, followed by the addition of benzyl bromide (41 mmol). The reaction mixture was refluxed and monitored by TLC until the starting material was consumed. The mixture was cooled to 0 °C, the solids were filtered off and washed with EtOAc, and the organic solvent was removed under reduced pressure. The residue was purified by flash chromatography on silica gel.

#### 1-Benzylpyrrolidine (**9**)

The crude reaction mixture was purified by flash chromatography on silica gel using hexanes/AcOEt (3:1) to give compound **9** (5.93 g, 92% yield) as a yellow oil. ^1^H NMR (500 MHz, CDCl_3_) δ: 7.34–7.22 (m, 5H), 3.61 (s, 2H), 2.53–2.48 (m, 4H), 1.81–1.75 (m, 4H). ^13^C{^1^H} NMR (126 MHz, CDCl_3_) δ: 139.5, 129.0, 128.3, 126.9, 60.8, 54.3, 23.5.

#### 1-Benzylpiperidine (**3**)

The crude reaction mixture was purified by flash chromatography on silica gel using hexanes/AcOEt (2:1) to give compound **3** (6.73 g, 96% yield) as a light yellow oil. ^1^H NMR (500 MHz, CDCl_3_) δ: 7.31–7.16 (m, 5H), 3.43 (s, 2H), 2.47–2.21 (m, 4H), 1.57–1.52 (m, 4H), 1.41–1.38 (m, 2H). ^13^C{^1^H} NMR (126 MHz, CDCl_3_) δ: 138.6, 129.0, 127.9, 126.7, 63.8, 54.4, 25.9, 24.3.

#### 1-Benzylazepane (**19**)

The crude reaction mixture was purified by flash chromatography on silica gel using hexanes/AcOEt (2:1) to give compound **19** (6.81 g, 90% yield) as a yellow oil. ^1^H NMR (500 MHz, CDCl_3_) δ: 7.32–7.14 (m, 5H), 3.59 (s, 2H), 2.59–2.54 (m, 4H), 1.62–1.55 (m, 8H). ^13^C{^1^H} NMR (126 MHz, CDCl_3_) δ: 140.0, 128.6, 128.0, 126.6, 62.8, 55.5, 28.3, 27.0.

#### 4-Methoxybenzylpiperidine (**17**)

The compound **17** was synthetized according to the procedure reported by Sartillo-Piscil et al.^12^

#### (*S*)-1-Benzyl-2-(((tert-butyldiphenylsilyl)oxy)methyl)pyrrolidine (**12**)

To a solution of imidazole (11.7 mmol) and *t*-Bu(Ph)_2_SiCl (9.4 mmol) in 39 mL of dry CH_2_Cl_2_ was added a solution of *N*-benzyl-*L*-prolinol (7.8 mmol) in dry CH_2_Cl_2_ (10 mL). The reaction mixture was stirred and monitored by TLC until the starting material was consumed, and then 5 mL of aqueous solution of NaHCO_3_ was added. Both phases were separated, and the aqueous phase was extracted with EtOAc (3 × 15 mL). The combined organic phases were dried over Na_2_SO_4_, and the solvent was removed under reduced pressure. The residue was purified by CC [SiO_2_, hexane/EtOAc, 14:1] to give 2.9 g (87%) of **12** as a colorless oil. ^1^H NMR (500 MHz, CDCl_3_) δ 7.69–7.67 (m, 4H), 7.39–7.33 (m, 6H), 7.28–7.22 (m, 4H), 7.18–7.15 (m, 1H), 4.08 (d, *J* = 13.1 Hz, 1H), 3.73 (dd, *J* = 10.1, 5.2 Hz, 1H), 3.56 (dd, *J* = 10.0, 6.4 Hz, 1H), 3.34 (d, *J* = 13.1 Hz, 1H), 2.88 (m, 1H), 2.74 (m, 1H), 2.17 (m, 1H), 1.92 (m, 1H), 1.65 (m, 3H) 1.06 (s, 9H). ^13^C{^1^H} NMR (126 MHz, CDCl_3_) δ: 140.0, 135.7, 135.7, 129.6, 128.9, 128.1, 127.7, 126.7, 67.8, 65.1, 59.9, 54.8, 28.5, 26.9, 23.0, 19.3.

### Method A

#### Dual C–H Oxidation with TEMPO/NaClO_2_/NaClO

The 3-alkoxyaminolactam precursors (**4a**, **4b**, **4c**, and **4d**) were prepared using the procedure developed by Sartillo-Piscil and co-workers^8^ To a mixture of NaH_2_PO_4_ (10 equiv), NaClO_2_ (3 equiv), and TEMPO (2 equiv) in CH_3_CN (5 mL) was added NaOCl (2.2 equiv) at 0 °C; then, the respective pyrrolidine or piperidine was added. The reaction mixture was stirred and monitored by TLC. Upon completion, a saturated aqueous solution of NaOH was added to the reaction mixture until the red-wine color was turned into orange. Then, EtOAc was added, and the phases were separated. The organic phase was washed with a solution of NH_4_Cl, and the aqueous phase was extracted with EtOAc. The combined organic phases were dried (Na_2_SO_4_), and the solvent was removed under reduced pressure. The residue was purified by CC.

##### (5*S*)-1-Benzyl-5-(((tert-butyldiphenylsilyl)oxy)methyl)-3-((2,2,6,6tetramethylpiperidin-1-yl)oxy)pyrrolidin-2-one (**4c**)

Compound **4c** was prepared following the general Method A using compound **12** (250.0 mg, 0.581 mmol) as starting material. The crude reaction was purified by flash chromatography on silica gel using hexanes/AcOEt (9:1) to give the 3-alkoxyamino lactam **4c** (174.2 mg, 50% yield) as a light yellow oil. ^1^H NMR (500 MHz, CDCl_3_) δ 7.62–7.57 (m, 4H), 7.47–7.36 (m, 6H), 7.26–7.21 (m, 3H), 7.06 (d, *J* = 6.7 Hz, 2H), 4.94 (d, *J* = 15.1 Hz, 1H), 4.61 (t, *J* = 8.2 Hz, 1H), 4.00 (d, *J* = 15.0 Hz, 1H), 3.66 (d, *J* = 4.7 Hz, 2H), 3.34–3.29 (m, 1H), 2.38 (m, 1H), 2.06–2.00 (m, 1H), 1.57 (br, 1H), 1.47 (br, 7H), 1.31 (br, 1H), 1.25 (s, 3H), 1.17 (br, 3H), 1.10 (s, 3H), 1.05 (s, 9H). ^13^C{^1^H} NMR (126 MHz, CDCl_3_) δ: 173.0, 136.8, 135.8, 135.7, 133.1, 130.0, 130.0, 128.7, 128.1, 128.0, 127.9, 127.4, 82.8, 64.3, 61.3, 59.3, 54.6, 44.9, 40.6, 40.5, 34.7, 32.8, 31.5, 26.9, 20.4, 20.3 19.3, 17.3.

##### 1-(4-Methoxybenzyl)-3-[(2,2,6,6-tetramethylpiperidin-1-yl)oxy]piperidin-2-one (**4d**)

The spectral properties of this compound are identical to those reported by Sartillo-Piscil et al.^12^

### Method B

#### Double C–H Oxidation with TEMPO Cation

To a solution of cyclic amine (pyrrolidine, piperidine, or azepane) (2.17 mmol) in CH_3_CN (72.3 mL) was added 2,2,6,6-tetramethyl-1-oxo-piperidinium tetrafluoroborate (6.5 mmol).^9^ Immediately, NaClO_2_ (7.6 mmol) was added. The mixture was allowed to stir for 15 min at rt. Finally, the reaction was quenched by adding brine until the red-wine color was turned into a clear-orange color. The resulting phases were separated, and the aqueous phase was extracted with AcOEt (4 × 15 mL). Organic portions were concentrated and purified by a flash chromatography.

##### 1-Benzyl-3-((2,2,6,6-tetramethylpiperidin-1-yl)oxy)azepan-2-one (**4e**)

Organic portions were concentrated and purified by flash chromatography on silica gel using hexanes/AcOEt (8:1) to give the 7 membered 3-alkoxyamino lactam (505.0 mg, 65% yield) as a white solid. Mp = 100–102 °C. ^1^H NMR (500 MHz, CDCl_3_) δ 7.31–7.12 (m, 5H), 4.62 (d, *J* = 15.5 Hz, 1H), 4.58 (d, *J* = 7.5 Hz, 1H)), 4.50 (d, *J* = 14.4 Hz, 1H), 3.95 (dd, *J* = 14.9, 10.8 Hz, 1H), 3.10 (dd, *J* = 14.9, 6.2 Hz, 1H), 2–26–2.20 (m, 1H), 1.94–1.86 (m, 1H), 1.65–1.57 (m, 4H), 1.51–1.46 (m, 4H), 1.30–1.10 (m, 14H). ^13^C{^1^H} NMR (126 MHz, CDCl_3_) δ: 174.8, 137.9, 128.9, 128.5, 127.4, 88.5, 60.0, 59.5, 51.4, 46.6, 40.2, 33.5, 29.8, 28.4, 27.9, 23.6, 21.3, 20.6, 17.3. HRMS (ESI-QTOF) *m*/*z*: [M+H]^+^ calcd for C_22_H_35_N_2_O_2_, 359.2693; found, 359. 2701.

#### β-Alkenylation (Olefination) of 3-Alkoxyamino Lactams

To a solution of 3-alkoxyamino lactam (0.580 mmol) and the respective phosphonium salt (1.74 mmol) in dry *t*-BuOH (11.6 mL) at rt was added 1.0 M potassium *t*-butoxide (4.06 mmol). Immediately a color change from colorless to pale yellow or orange was observed. The reaction mixture was stirred and monitored by TLC until the starting material is consumed. Finally, EtOAc (11.6 mL) and H_2_O (11.6 mL) were added. The resulting phases were separated; the aqueous phase was extracted with AcOEt (3 × 10 mL). Organic portions were concentrated and purified by flash chromatography on silica gel using hexanes/AcOEt as eluent.

##### Methyl (*E*)-2-(1-Benzyl-2-oxopiperidin-3-ylidene)acetate (***E-8*** and ***Z-8***)

The alkoxyamino lactam **4a** was used to obtain ***E-8***. The residue was purified by CC [SiO2, hexane/EtOAc, 2:1] to give 0.116 g (78% yield) of ***E-8*** as a colorless oil. ^1^H NMR (500 MHz, CDCl_3_) δ 7.27–7.18 (m, 5H), 6.97 (t, *J =* 2.2 Hz, 1H), 4.61 (s, 2H), 3.68 (s, 3H), 3.25 (apparent t, *J* = 5.8 Hz, 2H), 3.02 (td, *J* = 6.5, 2.1 Hz, 2H), 1.76 (quint, *J* = 6.25 Hz, 2H). ^13^C NMR (126 MHz, CDCl_3_) δ: 166.9, 163.1, 145.8, 136.7, 128.8, 128.1, 127.7, 123.5, 51.6, 51.5, 47.2, 26.4, 22.2. HRMS-ESI *m*/*z*: [M+H]^+^ calcd for C_15_H_18_NO_3_, 260.1281; found, 260.1281. Isolated 3.7 mg (2.6% yield) of ***Z-8*** as a colorless oil. ^1^H NMR (500 MHz, CDCl_3_) δ 7.25–7.17 (m, 5H), 5.95 (t, *J* = 1.8 Hz, 1H), 4.57 (s, 2H), 3.78 (s, 3H), 3.20–3.17 (apparent t, *J* = 6.0, Hz 2H), 2.51 (td, *J* = 6.4, 1.8 Hz, 2H), 1.82 (m, 2H). ^13^C{^1^H} NMR (126 MHz, CDCl_3_) δ: 168.7, 162.5, 136.8, 135.5, 128.8, 128.3, 127.7, 127.0, 52.4, 50.5, 47.3, 30.3, 22.7. HRMS (ESI-QTOF) *m*/*z*: [M+H]^+^ calcd for C_15_H_18_NO_3_, 260.1281; found, 260.1270.

##### Methyl (*E*)-2-(1-Benzyl-2-oxopyrrolidin-3-ylidene)acetate (**10**)

The alkoxyamino lactam **4b** was used to obtain **10**. The residue was purified by CC [SiO_2_, hexane/EtOAc, 2:1] to give 0.082 g (58% yield) of **10** as a colorless oil. ^1^H NMR (500 MHz, CDCl_3_) δ 7.37–7.22 (m, 5H), 6.67 (t, *J* = 3.1 Hz, 1H), 4.60 (s, 2H), 3.77 (s, 3H), 3.37 (t, *J* = 6.1 Hz, 2H), 3.16 (td, *J* = 6.1, 3.1 Hz, 2H). ^13^C{^1^H} NMR (126 MHz, CDCl_3_) δ: 166.9, 166.8, 149.4, 135.8, 129.0, 128.5, 128.1, 118.9, 51.8, 47.6, 44.4, 24.8. HRMS (ESI-QTOF) *m*/*z*: [M + H]^+^ calcd for C_14_H_16_NO_3,_ 246.1125; found, 246.1125.

##### (*E*)-1-Benzyl-3-(2-oxopropylidene)pyrrolidin-2-one (**11**)

The alkoxyamino lactam **4b** (10.0 mg, 0.03 mmol) was used to obtain **11**. The residue was purified by CC [SiO_2_, hexane/EtOAc, 2:1] to give 2.7 mg (40% yield) of **11** as a colorless oil. ^1^H NMR (500 MHz, CDCl_3_) δ 7.36–7.30 (m, 5H), 7.01 (t, *J* = 3.1 Hz, 1H), 4.62 (s, 2H), 3.36 (t, *J* = 6.0 Hz, 2H), 3.13 (td, *J* = 6.0, 3.1 Hz, 2H), 2.34 (s, 3H). ^13^C{^1^H} NMR (126 MHz, CDCl_3_) δ: 199.1, 167.5, 147.1, 135.9, 129.0, 128.5, 128.1, 124.3, 47.7, 44.6, 29.9, 25.4. HRMS (ESI-QTOF) *m*/*z*: [M + H]^+^ calcd for C_14_H_16_NO_2_ 230.1176; found, 230.1151.

##### Methyl (*S*,*E*)-2-(1-Benzyl-5-(((tert-butyldiphenylsilyl)oxy)methyl)-2-oxopyrrolidin-3-ylidene)acetate (**13**)

The alkoxyamino lactam **4c** was used to obtain **13**. The residue was purified by CC [SiO_2_, hexane/EtOAc, 6:1] to give 0.201 g (68% yield) of **13** as a colorless oil. ^1^H NMR (500 MHz, CDCl_3_) δ 7.52–7.49 (m, 4H), 7.40–7.30 (m, 6H), 7.19–7.15 (m, 3H), 7.00–6.98 (m, 2H), 6.62 (t, *J* = 3.0 Hz, 1H), 5.03 (d, *J* = 14.5 Hz, 1H), 3.71 (s, 3H), 3.70 (d, *J* = 15.0 Hz, 1H), 3.63 (dd, *J* = 7.9, 3.9 Hz, 1H), 3.50 (m, 2H), 3.17 (d, *J* = 20.0 Hz, 1H), 2.99 (ddd, *J* = 20.0, 7.8, 3.3 Hz, 1H), 0.93 (s, 9H). ^13^C{^1^H} NMR (126 MHz, CDCl_3_) δ: 167.4, 166.9, 149.5, 135.8, 135.7, 135.6, 132.7, 132.7, 130.1, 130.0, 128.8, 128.2, 127.9, 127.9, 127.7, 118.2, 62.8, 55.9, 51.7, 44.9, 29.0, 26.7, 19.1. HRMS (ESI-QTOF) *m*/*z*: [M + H]^+^ calcd for C_31_H_36_NO_4_Si, 514.2408; found, 514.2416.

##### (*S*,*E*)-1-Benzyl-5-(((tert-butyldiphenylsilyl)oxy)methyl)-3-(2-oxopropylidene)pyrrolidin-2-one (**14**)

The alkoxyamino lactam **4c** was used to obtain **14**. The residue was purified by CC [SiO_2_, hexane/EtOAc, 6:1] to give 0.236 g (48% yield) of **14** as a colorless oil. ^1^H NMR (500 MHz, CDCl_3_) δ 7.51–7.49 (m, 4H), 7.38–7.30 (m, 6H), 7.20–7.15 (m, 3H), 7.00–6.99 (m, 2H), 6.83 (d, *J* = 1.5 Hz, 1H), 4.94 (d, *J* = 15.2 Hz, 1H), 4.03 (d, *J* = 15.3 Hz, 1H), 3.92 (apparent t, *J* = 4.4 Hz, 1H), 3.74 (dd, *J* = 10.5, 4.7 Hz, 1H), 3.57 (dd, *J* = 10.5, 5.8 Hz, 1H), 3.45 (d, *J* = 17.4 Hz, 1H), 3.36 (d, *J* = 17.4 Hz, 1H), 2.18 (s, 3H), 0.95 (s, 9H). ^13^C{^1^H} NMR (126 MHz, CDCl_3_) δ: 204.7, 171.4, 142.1, 137.4, 135.7, 135.7, 132.9, 132.8, 132.7, 130.1, 130.1, 128.8, 128.0, 128.0, 127.9, 127.5, 63.1, 62.0, 44.8, 39.9, 30.4, 26.9, 19.3. HRMS (ESI-QTOF) *m*/*z*: [M + H]^+^ calcd for C_31_H_36_NO_3_Si, 498.2459; found, 498.2462.

##### (*E*)-1-Benzyl-3-(2-oxopropylidene)piperidin-2-one (**15**)

The alkoxyamino lactam **4a** was used to obtain **15**. The residue was purified by CC [SiO_2_, hexane/EtOAc, 2:1] to give 75.0 mg (54% yield) of **15** as a colorless oil. ^1^H NMR (500 MHz, CDCl_3_) δ 7.29–7.18 (m, 6H), 4.61 (s, 2H), 3.26 (t, *J* = 5.7 Hz, 2H), 2.95 (t, *J* = 6.5 Hz, 2H), 2.25 (s, 3H), 1.74 (quint, *J* = 6.0 Hz, 2H). ^13^C{^1^H} NMR (126 MHz, CDCl_3_) δ: 199.9, 163.6, 142.9, 136.7, 129.2, 128.8, 128.1, 127.7, 51.6, 47.1, 32.5, 26.6, 22.2. HRMS (ESI-QTOF) *m*/*z*: [M + H]^+^ calcd for C_15_H_18_NO_2,_ 244.1332; found, 244.1337.

##### (*E*)-1-Benzyl-3-(2-oxo-2-phenylethylidene)piperidin-2-one (**16**)

The alkoxyamino lactam **4a** was used to obtain **16**. The residue was purified by column chromatography [SiO_2_, hexane/EtOAc, 3:1] to give 0.107 g (61% yield) of **16** as a colorless oil. ^1^H NMR (500 MHz, CDCl_3_) δ 8.09 (t, *J* = 2.2 Hz, 1H), 8.04 (d, *J* = 7.0 Hz, 2H), 7.58 (t, *J* = 7.4 Hz, 1H), 7.49 (t, *J* = 7.7 Hz, 2H), 7.36–7.26 (m, 5H), 4.74 (s, 2H), 3.37 (t, *J* = 5.8 Hz, 2H), 3.04 (td, *J* = 6.7, 2.2 Hz, 2H), 1.86 (quint, *J* = 6.0 Hz, 2H). ^13^C{^1^H} NMR (126 MHz, CDCl_3_) δ: 192.5, 163.7, 143.8, 138.2, 136.8, 133.4, 128.9, 128.8, 128.7, 128.2, 128.0, 127.7, 51.7, 47.4, 27.1, 22.4. HRMS (ESI-QTOF) *m*/*z*: [M + H]^+^ calcd for C_20_H_20_NO_2,_ 306.1489; found, 306.1461.

##### (*E*)-1-(4-Methoxybenzyl)-3-(2-oxo-2-phenylethylidene)piperidin-2-one (**18**)

The alkoxyamino lactam **4d** was used to obtain **18**. The residue was purified by CC [SiO_2_, hexane/EtOAc, 3:1] to give 0.10 g (52% yield) of **18** as a colorless oil. ^1^H NMR (500 MHz, CDCl_3_) δ 8.08 (s, 1H), 8.03 (d, *J* = 7.5 Hz, 2H), 7.59 (t, *J* = 7.4 Hz, 1H), 7.49 (t, *J* = 7.7 Hz, 2H), 7.25 (d, *J* = 8.6 Hz, 2H), 6.88 (d, *J* = 8.6 Hz, 2H), 4.66 (s, 2H), 3.81 (s, 3H), 3.36 (t, *J* = 5.9 Hz, 2H), 3.02 (td, *J* = 6.9, 3.7 Hz, 2H), 1.84 (quint, *J* = 5.5 Hz, 2H). ^13^C{^1^H} NMR (126 MHz, CDCl_3_) δ: 192.6, 163.6, 159.3, 143.9, 138.2, 133.5, 129.7, 129.0, 128.8, 128.8, 127.9, 114.2, 55.4, 51.1, 47.2, 27.1, 22.5. HRMS (ESI-QTOF) *m*/*z*: [M + H]^+^ calcd for C_21_H_22_NO_3,_ 336.1594, found: 336.1572.

##### Methyl (*E*)-2-(1-Benzyl-2-oxoazepan-3-ylidene)acetate (**20**)

The alkoxyamino lactam **4e** was used to obtain **20**. The residue was purified by CC [SiO_2_, hexane/EtOAc, 3:1] to give 79.0 mg (50% yield) of **20** as a colorless oil. ^1^H NMR (500 MHz, CDCl_3_) δ 7.28–7.18 (m, 5H), 6.13 (s, 1H), 4.55 (s, 2H), 3.67 (s, 3H), 3.20–3.14 (m, 2H), 2.80 (apparent t, *J* = 5.3 Hz, 2H), 1.70 (quint, *J* = 6.1 Hz, 2H), 1.44 (m, 2H). ^13^C{^1^H} NMR (126 MHz, CDCl_3_) δ: 171.4, 166.4, 157.8, 137.2, 128.8, 128.4, 127.8, 120.1, 51.6, 50.5, 47.3, 28.1, 26.6, 25.6. HRMS (ESI-QTOF) *m*/*z*: [M + H]^+^ calcd for C_16_H_20_NO_3,_ 274.1438; found, 274.1442.

##### 1-(4-Methoxybenzyl)-3-(2-oxo-2-phenylethyl)-5,6-dihydropyridin-2(1*H*)-one (**21**)

To a solution of **18** (20.0 mg, 0.05 mmol) in CH_2_Cl_2_ (1.2 mL) was added TiCl_4_ dropwise (0.298 mL, 0.298 mmol). The resulting reaction mixture was stirred at rt overnight. When the starting material was completely consumed, 5 mL of H_2_O was added. The phases were separated, and the aqueous phase was extracted with CH_2_Cl_2_ (2 × 5 mL). The combined organic phases were dried over Na_2_SO_4_, and the solvent was evaporated under reduced pressure. The residue was purified by CC (SiO_2_, hexanes/EtOAc, 3:1) to give 14.0 mg (70% yield) of **21** as a colorless oil. ^1^H NMR (500 MHz, CDCl_3_) δ 8.04 (d, *J* = 8.2 Hz, 2H), 7.56 (t, *J* = 7.4 Hz, 1H), 7.47 (t, *J* = 7.8 Hz, 2H), 7.21 (d, *J* = 8.5 Hz, 2H), 6.86 (d, *J* = 8.7 Hz, 2H), 6.43 (t, *J* = 4.4 Hz, 1H), 4.58 (s, 2H), 4.00 (s, 2H), 3.80 (s, 3H), 3.36 (t, *J* = 7.2 Hz, 2H), 2.35 (m, 2H). ^13^C{^1^H} NMR (126 MHz, CDCl_3_) δ: 198.1, 164.7, 159.1, 137.2, 136.9, 133.2, 130.3, 129.6, 129.4, 128.7, 128.6, 114.1, 55.4, 49.6, 44.7, 40.4, 24.2. HRMS (ESI-QTOF) *m*/*z*: [M + H]^+^ calcd for C_21_H_22_NO_3,_ 336.1594; found, 336.1583.

##### 3-(2-Oxo-2-phenylethyl)-5,6-dihydropyridin-2(1*H*)-one (Callylactam A)

To a solution of **21** (0.01 g, 0.029 mmol) in MeCN (0.596 mL) at 0 °C was added a cold solution of CAN (0.04 g, 0.089 mmol) in H_2_O (0.50 mL). The reaction mixture was stirred for 6 h at 0 °C and then warmed to rt, and H_2_O (1.5 mL) was added. The resulting phases were separated, and the aqueous phase was extracted with EtOAc (3 × 3 mL). The combined organic layers were dried (Na_2_SO_4_) and concentrated under reduced pressure. The residue was purified by CC (SiO_2_, hexanes/EtOAc, 1:2) to give 4.74 mg (79% yield) of callylactam A as a colorless oil. ^1^H NMR (500 MHz, CDCl_3_) δ 8.01 (d, *J* = 7.2 Hz, 2H), 7.55 (t, *J* = 7.4 Hz, 1H), 7.45 (t, *J* = 7.7 Hz, 2H), 6.52 (t, *J* = 4.4 Hz, 1H), 5.54 (br, 1H), 3.96 (s, 2H), 3.48 (td, *J* = 7.1, 2.5 Hz, 2H), 2.43 (m, 2H). ^13^C{^1^H} NMR (126 MHz, CDCl_3_) δ: 197.8, 166.2, 139.1, 136.8, 133.3, 129.7, 128.7, 128.6, 40.1, 39.7, 24.6. For comparative purposes, ^1^H and ^13^C NNMR spectra were also recorded in CD_3_OD. ^1^H NMR (500 MHz, CD_3_OD) δ 8.01 (dd, *J* = 8.0, 1.2 Hz, 2H), 7.60 (t, *J* = 7.5 Hz, 1H), 7.49 (t, *J* = 7.0 Hz, 2H), 6.60 (t, *J* = 4.0 Hz, 1H), 3.97 (s, 2H), 3.42 (t, *J* = 7.0 Hz, 2H), 2.41 (q, *J* = 7.0 Hz, 2H). ^13^C{^1^H} NMR (126 MHz, CD_3_OD) δ: 199.8, 168.3, 141.3, 138.1, 134.3, 130.8, 129.7, 129.3, 41.1, 40.3, 25.1 The spectral properties of this compound are in agreement with isolated natural product.^10^

## Data Availability

The data underlying this study are available in the published article and its Supporting Information.

## Supporting Information Available

^1^H and ^13^C NMR spectra for new and relevant compounds (PDF)
