# Supplementary material for: β-Alkenylation of Saturated N-Heterocycles via a C(sp3)–O Bond Wittig-like Olefination
Source: J Org Chem. 2024 Jan 12;89(3):1762–8. doi: 10.1021/acs.joc.3c02466 (PMC10845111; doi:10.1021/acs.joc.3c02466)
Supplement: Supplementary file 1 — jo3c02466_si_001.pdf [file jo3c02466_si_001.pdf]

β-Alkenylation of Saturated *N*- Heterocycles Via a C(sp^3)−O Bond Wittig-Like Olefination

*Ángel A. Nolasco-Hernández, Leticia Quintero, Silvano Cruz-Gregorio and Fernando Sartillo-Piscil **

Centro de Investigación de la Facultad de Ciencias Químicas, Benemérita Universidad Autónoma de Puebla (BUAP), 14 Sur Esq. San Claudio, Col. San Manuel, 72570, Puebla, México.

fernando.sartillo@correo.buap.mx. Fax: +52222 2454972; Tel: +52 222 2955500 ext. 7391.

TABLE OF CONTENTS

General considerations SI-3

Gram scale synthesis of *E-8*......SI-4

^{1}H and ^{13}C NMR Spectra Compound 9....SI-5

^{1}H and ^{13}C NMR Spectra Compound 3....SI-6

^{1}H and ^{13}C NMR Spectra Compound 19....SI-7

^{1}H and ^{13}C NMR Spectra Compound 17....SI-8

^{1}H and ^{13}C NMR Spectra Compound 12....SI-9

^{1}H and ^{13}C NMR Spectra Compound 4a....SI-10

^{1}H and ^{13}C NMR Spectra Compound 4b....SI-11

^{1}H and ^{13}C NMR Spectra Compound 4c....SI-12

^{1}H and ^{13}C NMR Spectra Compound 4d....SI-13

^{1}H and ^{13}C NMR Spectra Compound 4e....SI-14

^{1}H and ^{13}C NMR Spectra Compound *E-8*....SI-15

^{1}H and ^{13}C NMR Spectra Compound *Z*-8....SI-16

^{1}H and ^{13}C NMR Spectra Compound 10....SI-17

^{1}H and ^{13}C NMR Spectra Compound 11....SI-18

^{1}H and ^{13}C NMR Spectra Compound 13....SI-19

^{1}H and ^{13}C NMR Spectra Compound 14....SI-20

^{1}H and ^{13}C NMR Spectra Compound 15....SI-21

^{1}H and ^{13}C NMR Spectra Compound 16....SI-22

^{1}H and ^{13}C NMR Spectra Compound 18....SI-23

^{1}H and ^{13}C NMR Spectra Compound 20....SI-24

^{1}H and ^{13}C NMR Spectra Compound 21....SI-25

^{1}H and ^{13}C NMR Spectra of Callylactam A........SI-26

^{1}H and ^{13}C NMR (CD_3OD) Spectra of Callylactam A....SI-27

References....SI-28

General considerations

General considerations: Unless otherwise stated, ^{1}H NMR and ^{13}C NMR spectra were obtained in a 500 MHz and 125 MHz spectrometer, respectively. All samples were analyzed in $CDCl_3$ with TMS as internal reference using a relative scale in parts per million (ppm) for the chemical shift (δ) and Hz for coupling constants (*J*). Splitting patterns are designated as follow: s, singlet; d, doublet; t, triplet; q, quartet; quint, quintet; m, multiple; and br, broad.

Grame scale synthesis of *E-8*.

To a solution of 3-alkoxyamino lactam **4a** (1.8 g, 5.2 mmol) and (Methoxycarbonylmethyl)triphenylphosphonium bromide (6.5 g, 15.6 mmol) in dry *t*-BuOH (104 mL) at room temperature was added a solution 1.0 M of potassium *t*-butoxide (36.5 mL, 36.5 mmol). Immediately a color change from colorless to pale yellow was observed. The reaction mixture was stirred over 8 h. Finally, EtOAc (50 mL) and H_2O (11.6 mL) were added. The resulting phases were separated; the aqueous phase was extracted with AcOEt (5×20 mL). Organic portions were concentrated and purified by flash chromatography [SiO_2, hexane/EtOAc, 3:1] to give 1.04 g (77%) of ***E-8*** as a colorless oil.

Visual details of gram scale synthesis of *E*-8

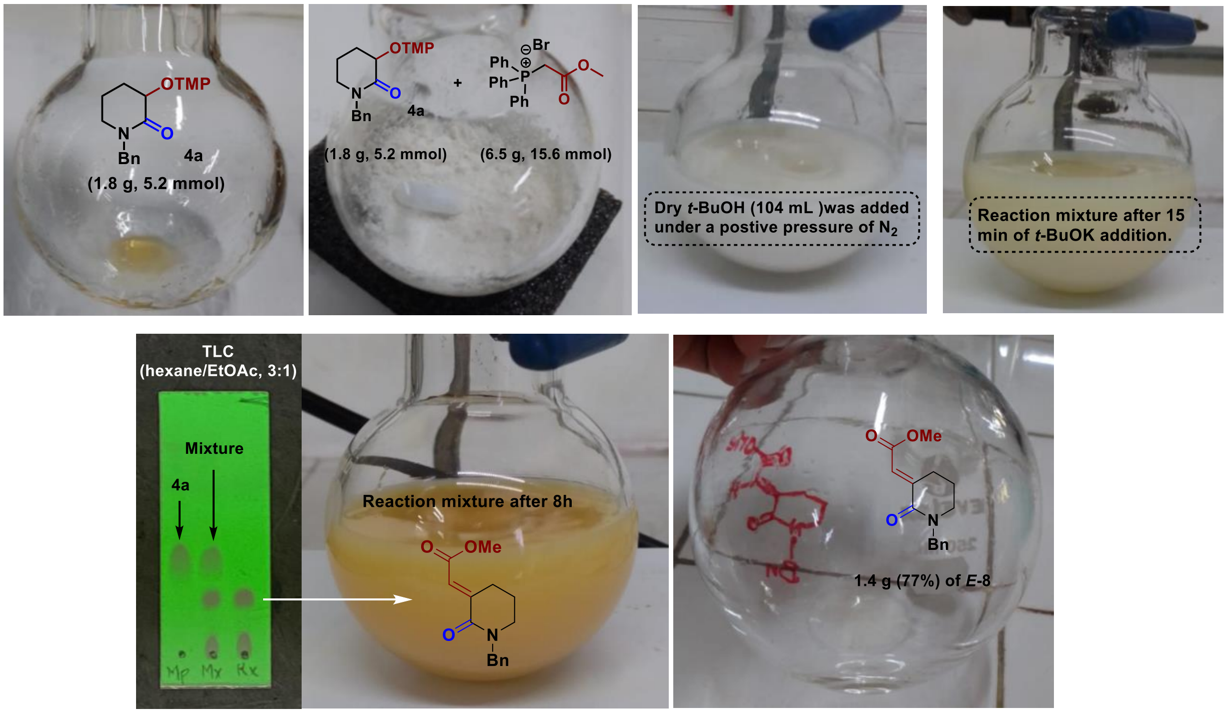

^{1}H NMR Spectrum of Compound 9 (500 MHz, $CDCl_3$)

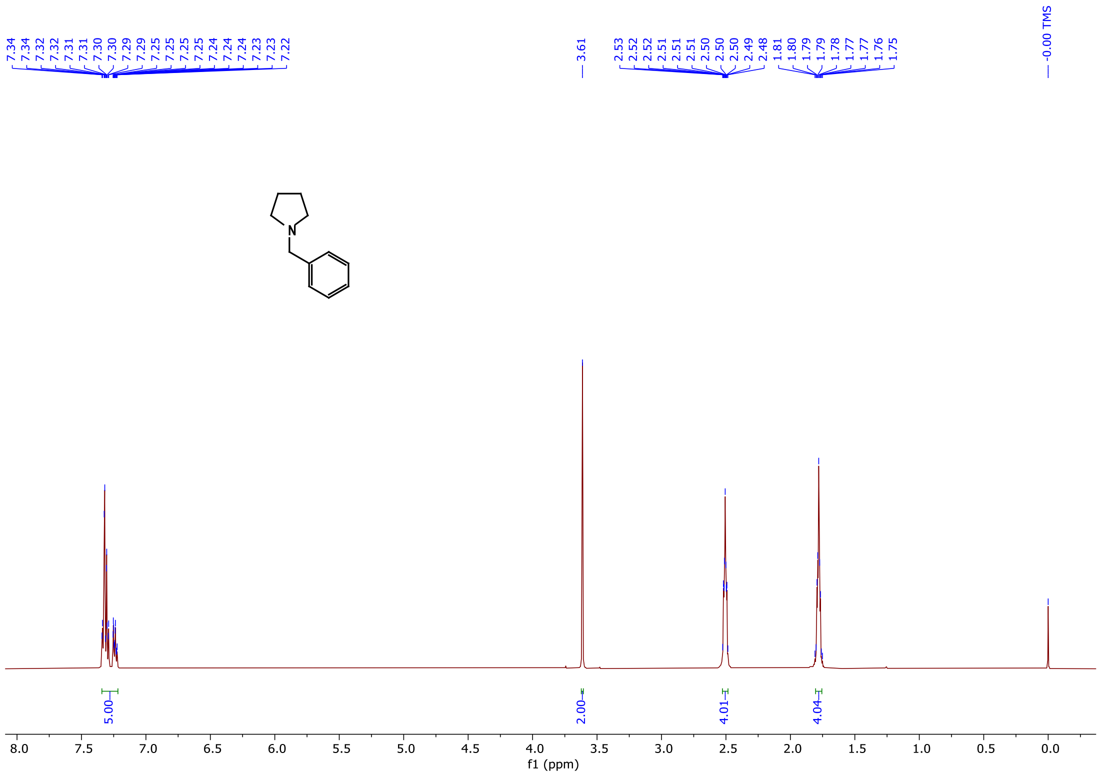

$^{13}C\{1H\}$ NMR Spectrum of Compound 9 (126 MHz, $CDCl_3$)

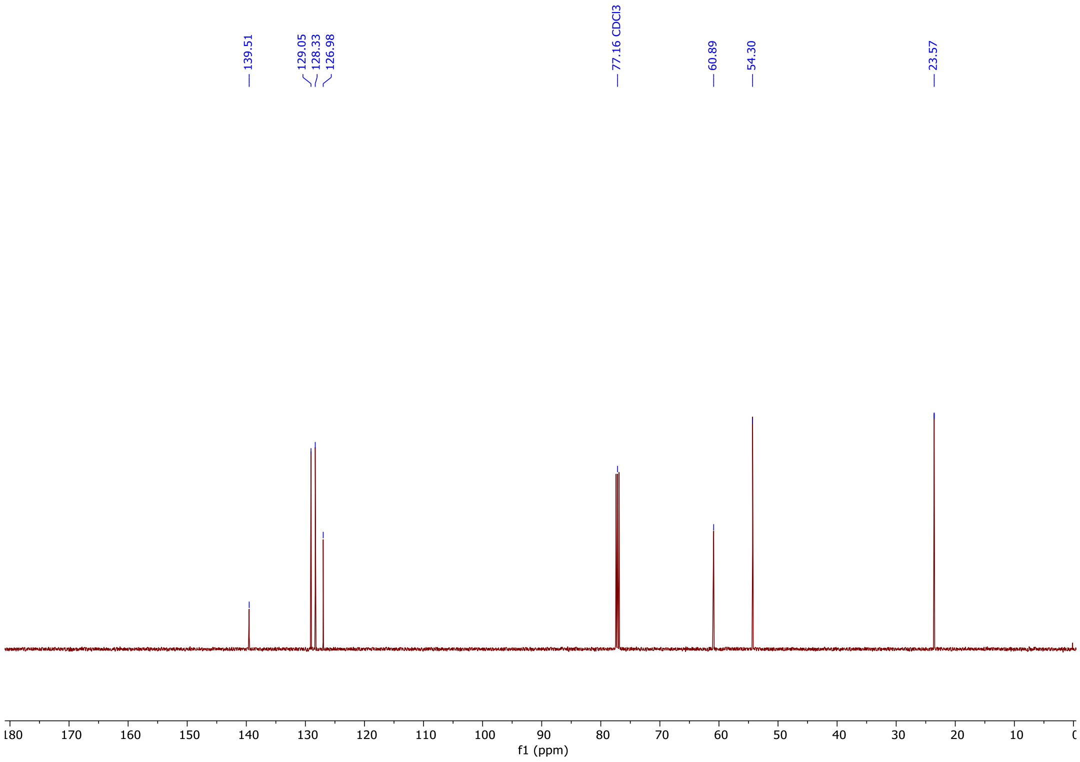

^{1}H NMR Spectrum of Compound 3 (500 MHz, $CDCl_3$)[1]

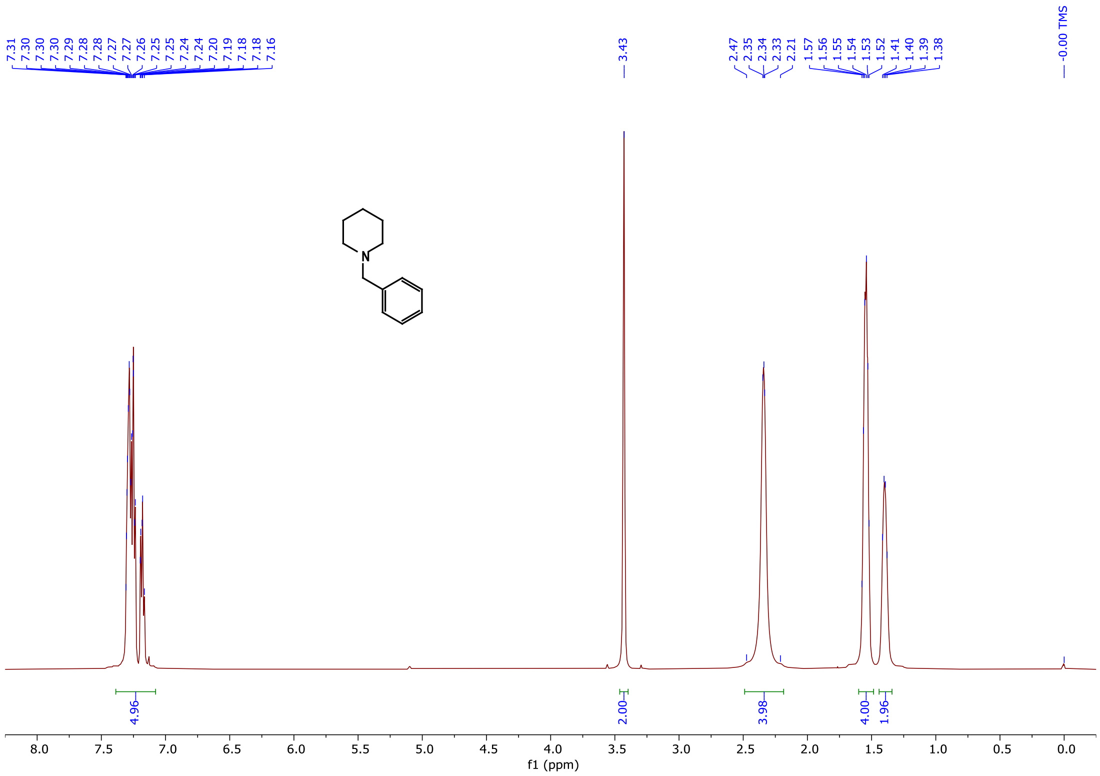

^{13}C{1H} NMR Spectrum of Compound 3 (126 MHz, $CDCl_3$)

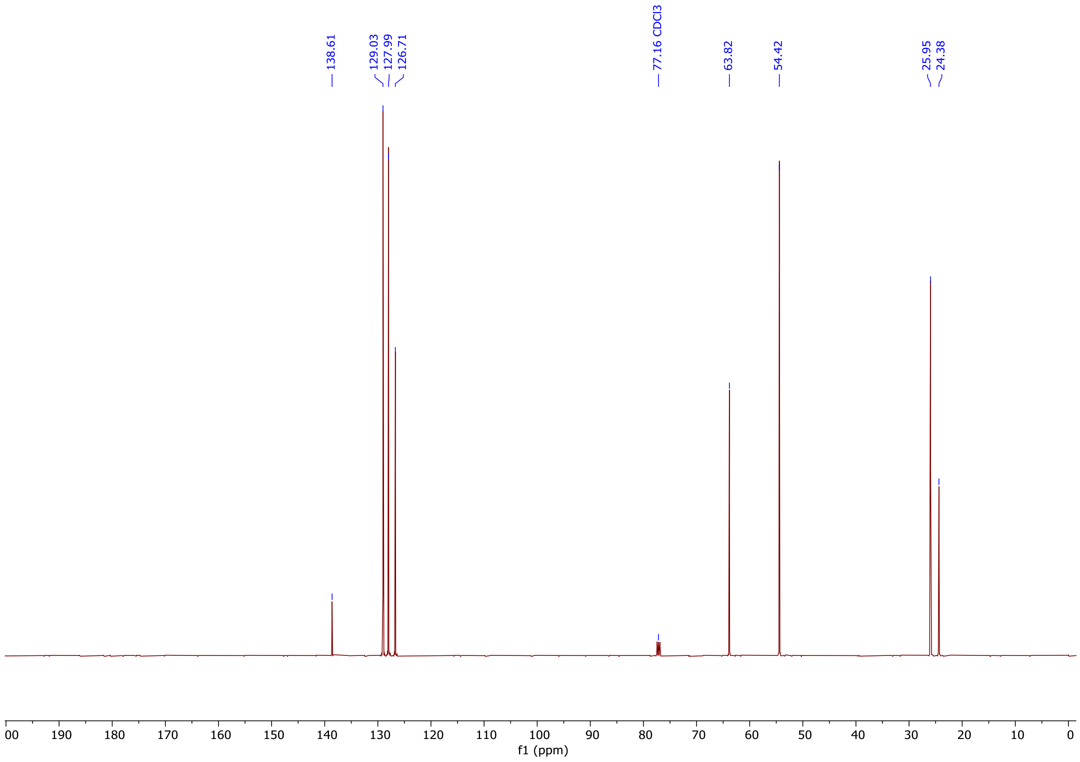

^{1}H NMR Spectrum of Compound 19 (500 MHz, $CDCl_3$)[1]

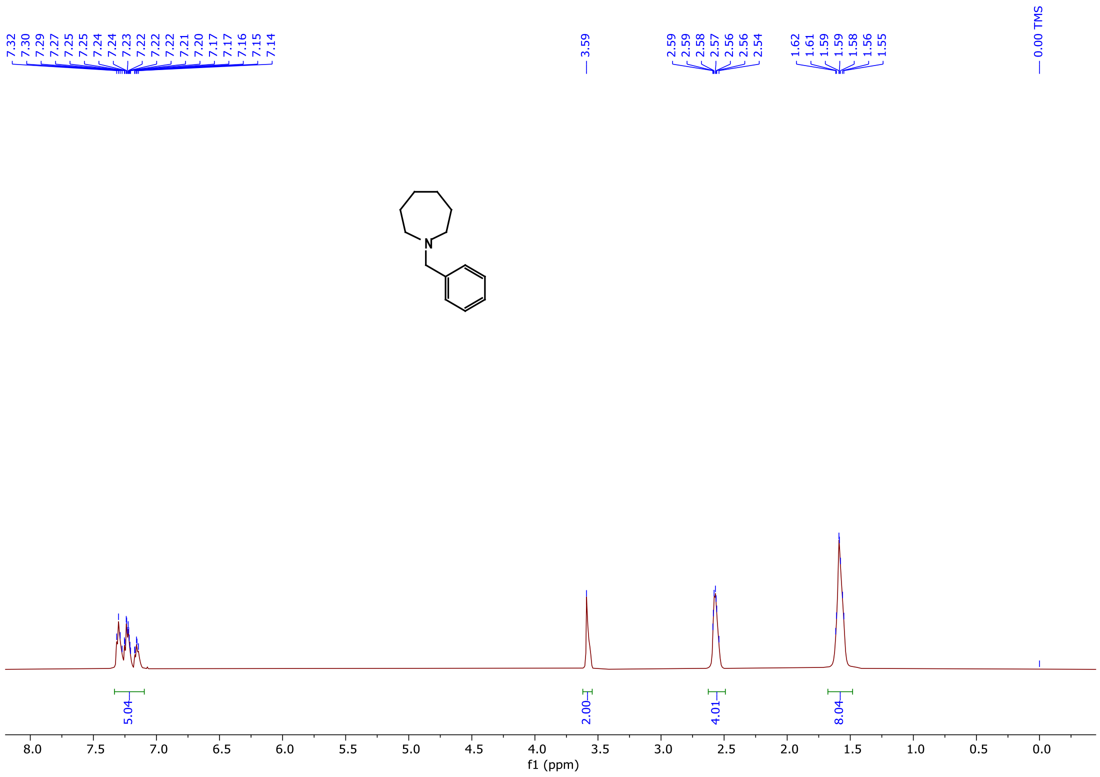

^{13}C{1H} NMR Spectrum of Compound 19 (126 MHz, $CDCl_3$)

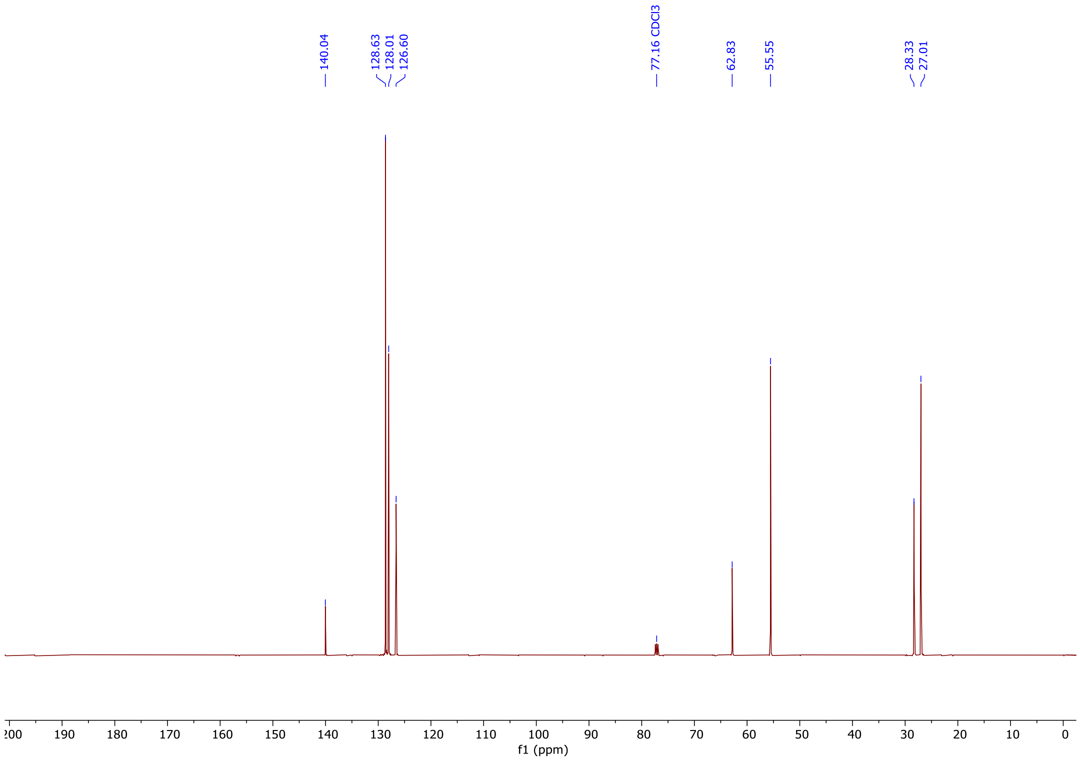

^{1}H NMR Spectrum of Compound 17 (500 MHz, $CDCl_3$)[2]

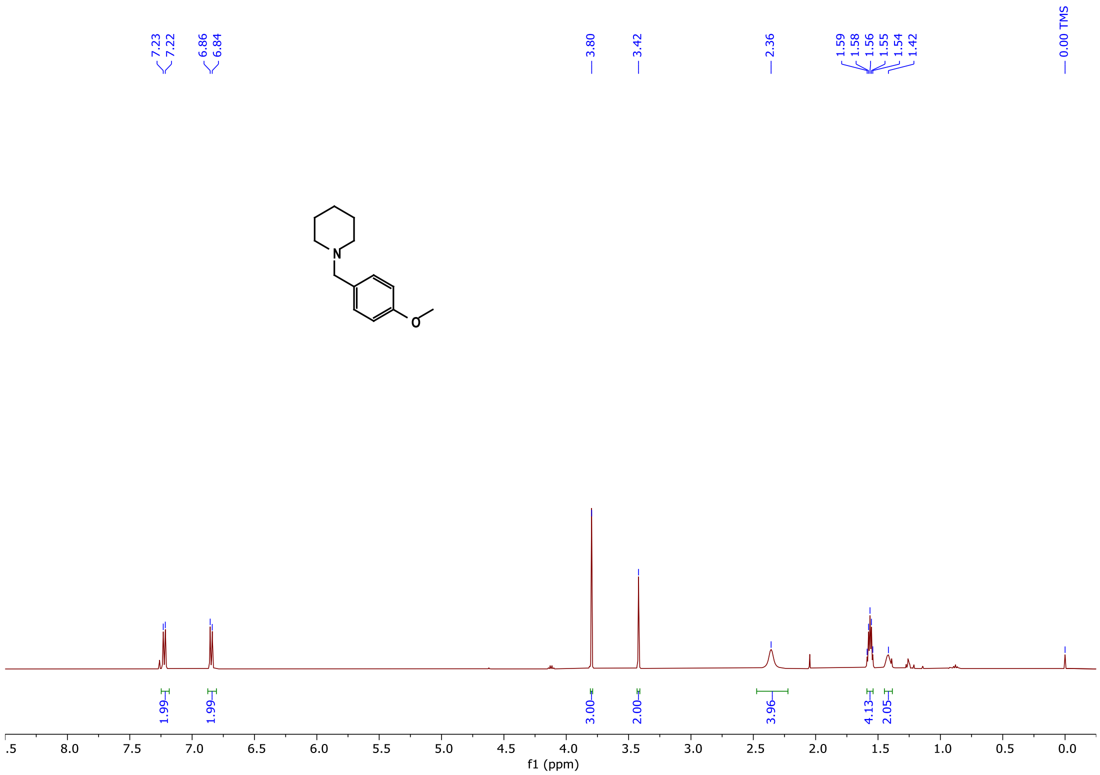

^{13}C{1H} NMR Spectrum of Compound 17 (126 MHz, $CDCl_3$)

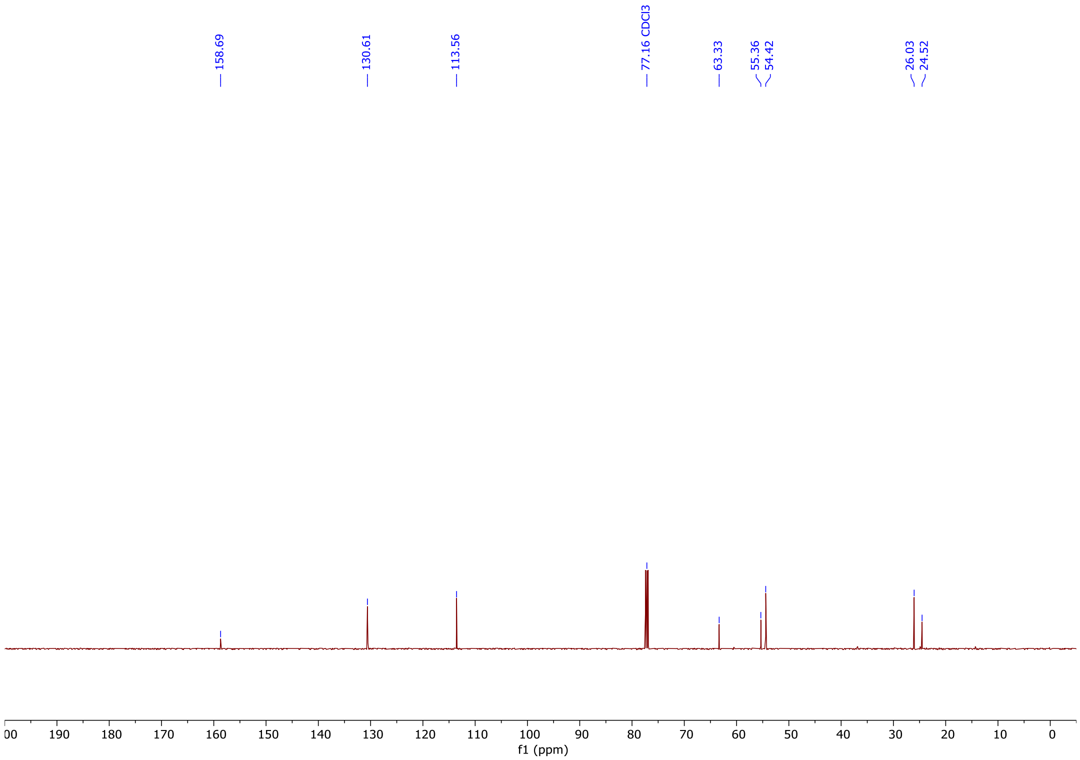

^{1}H NMR Spectrum of Compound 12 (500 MHz, $CDCl_3$)

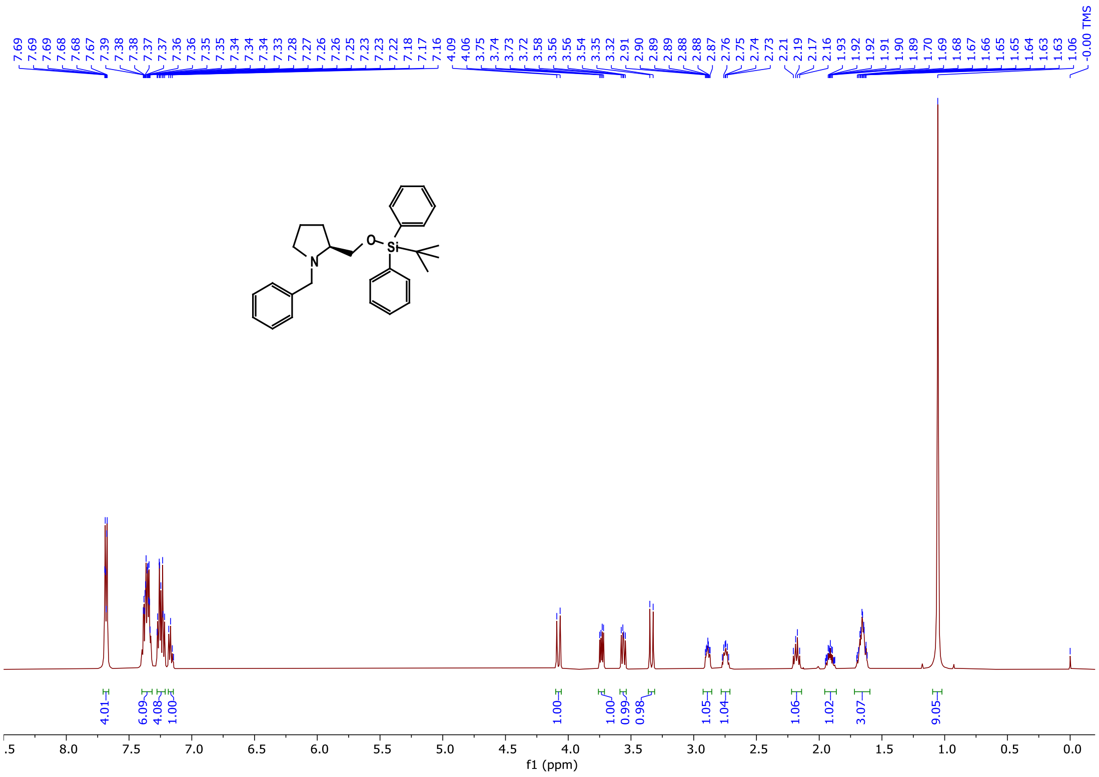

^{13}C{1H} NMR Spectrum of Compound 12 (126 MHz, $CDCl_3$)

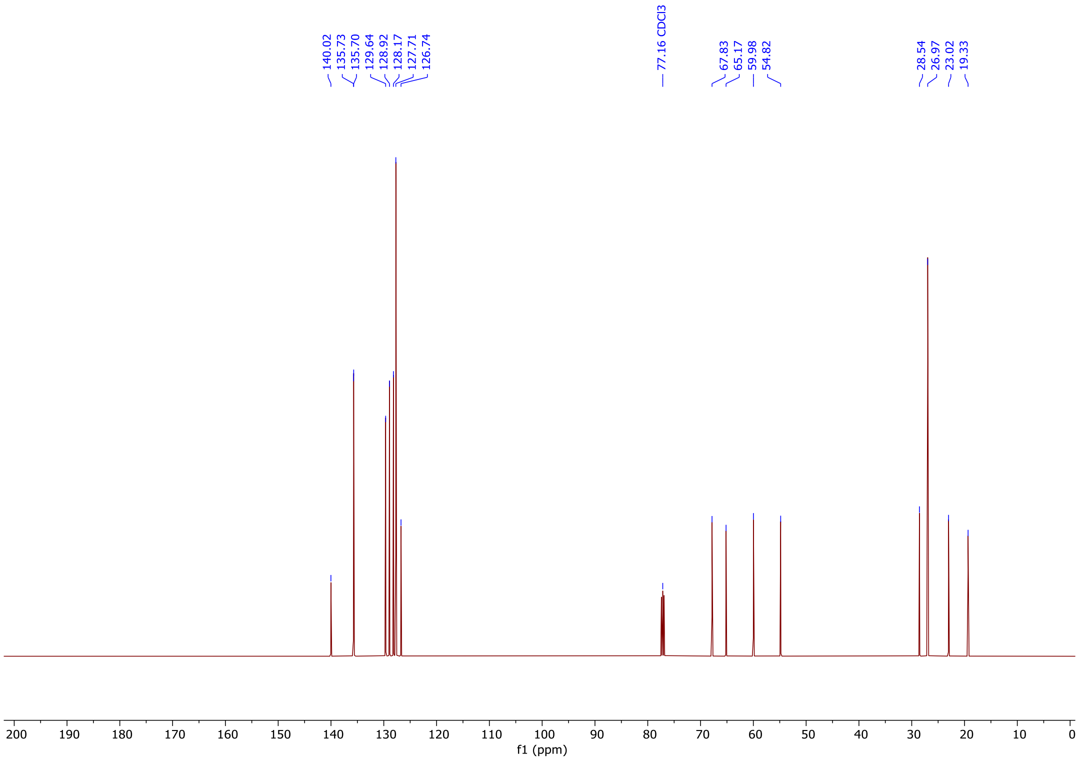

^{1}H NMR Spectrum of Compound 4a (500 MHz, $CDCl_3$)[3]

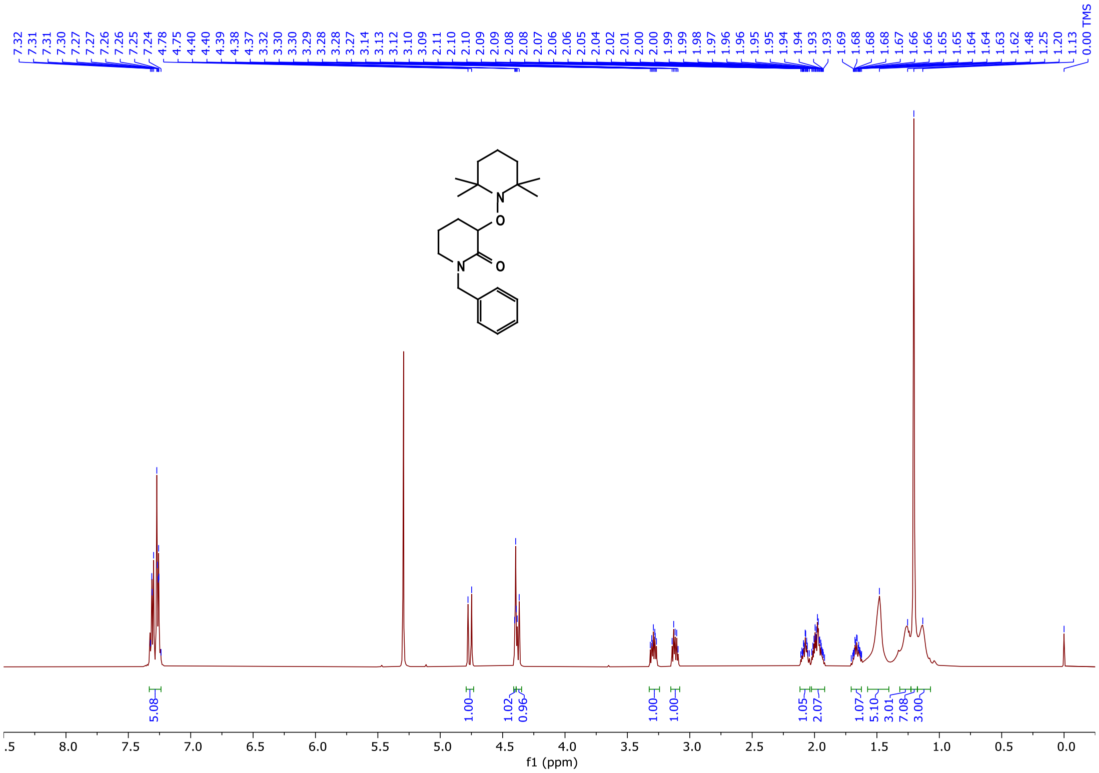

^{13}C{1H} NMR Spectrum of Compound 4a (126 MHz, $CDCl_3$)

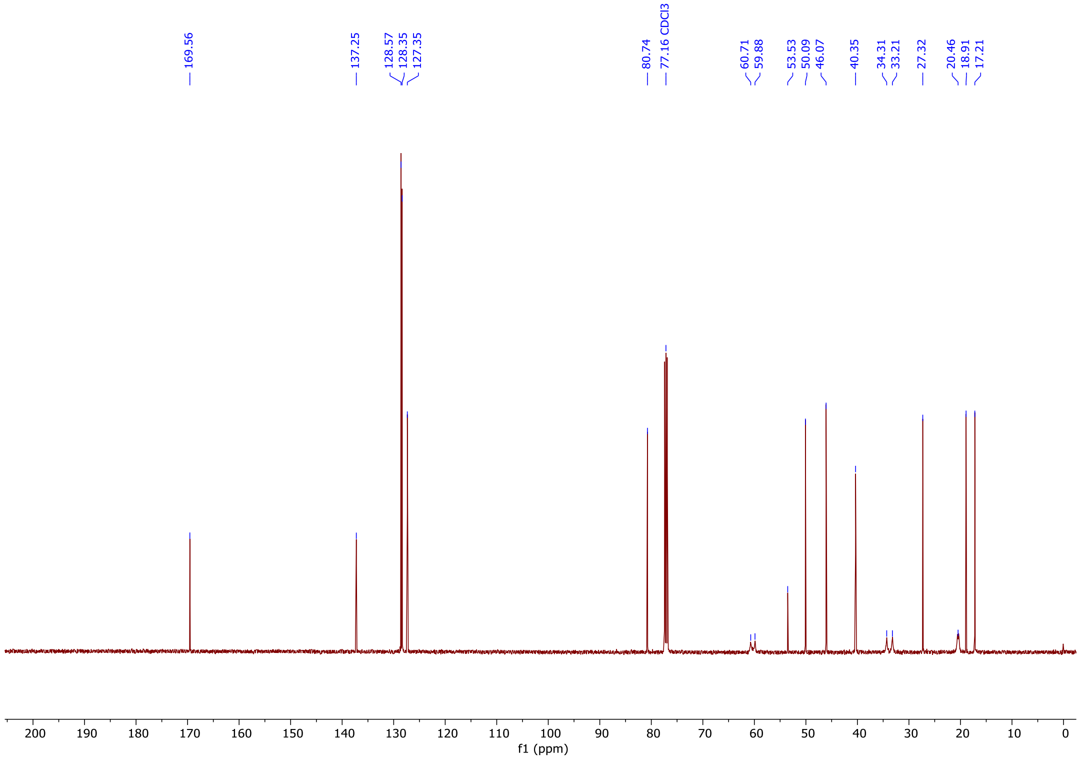

^{1}H NMR Spectrum of Compound 4b (500 MHz, $CDCl_3$)[3]

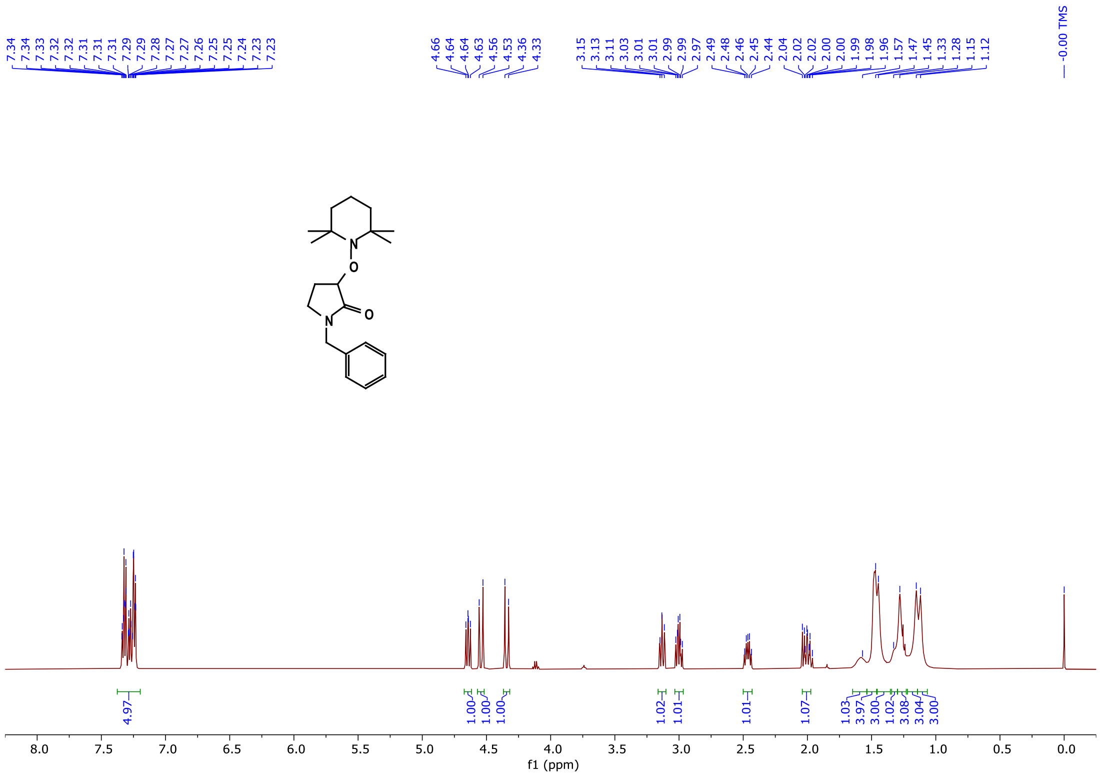

^{13}C{1H} NMR Spectrum of Compound 4b (126 MHz, $CDCl_3$)

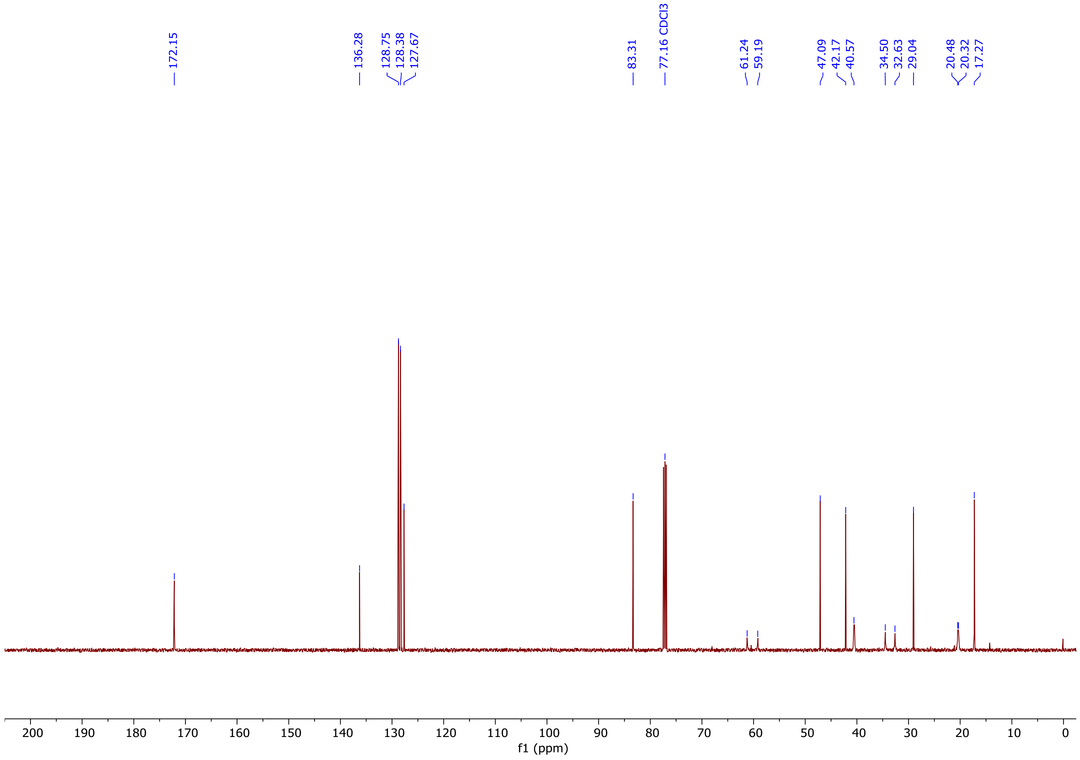

^{1}H NMR Spectrum of Compound 4c (500 MHz, $CDCl_3$)

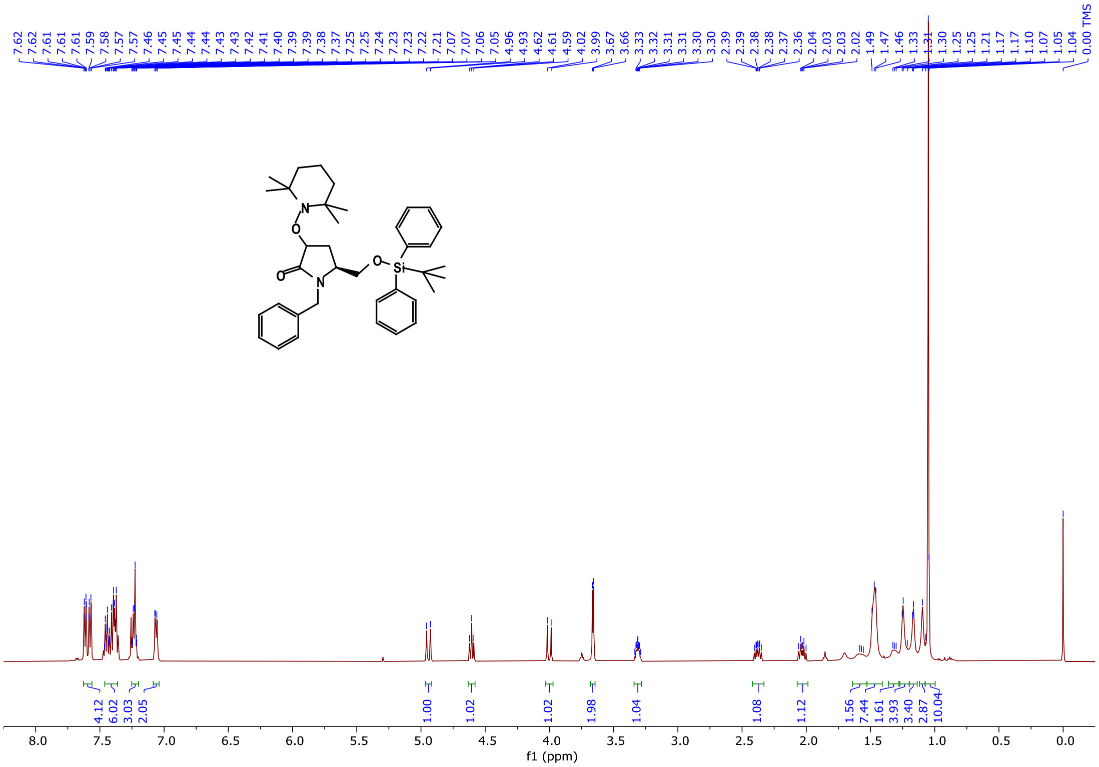

^{13}C{1H} NMR Spectrum of Compound 4c (126 MHz, $CDCl_3$)

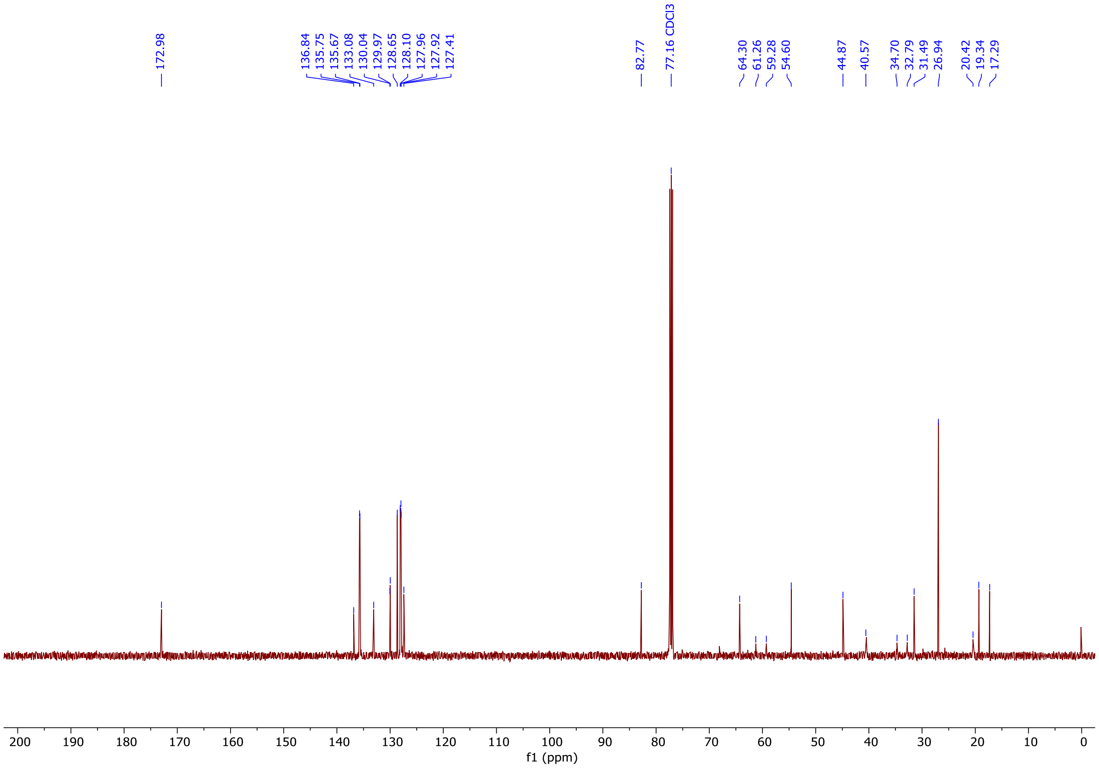

^{1}H NMR Spectrum of Compound 4d (500 MHz, $CDCl_3$)[2]

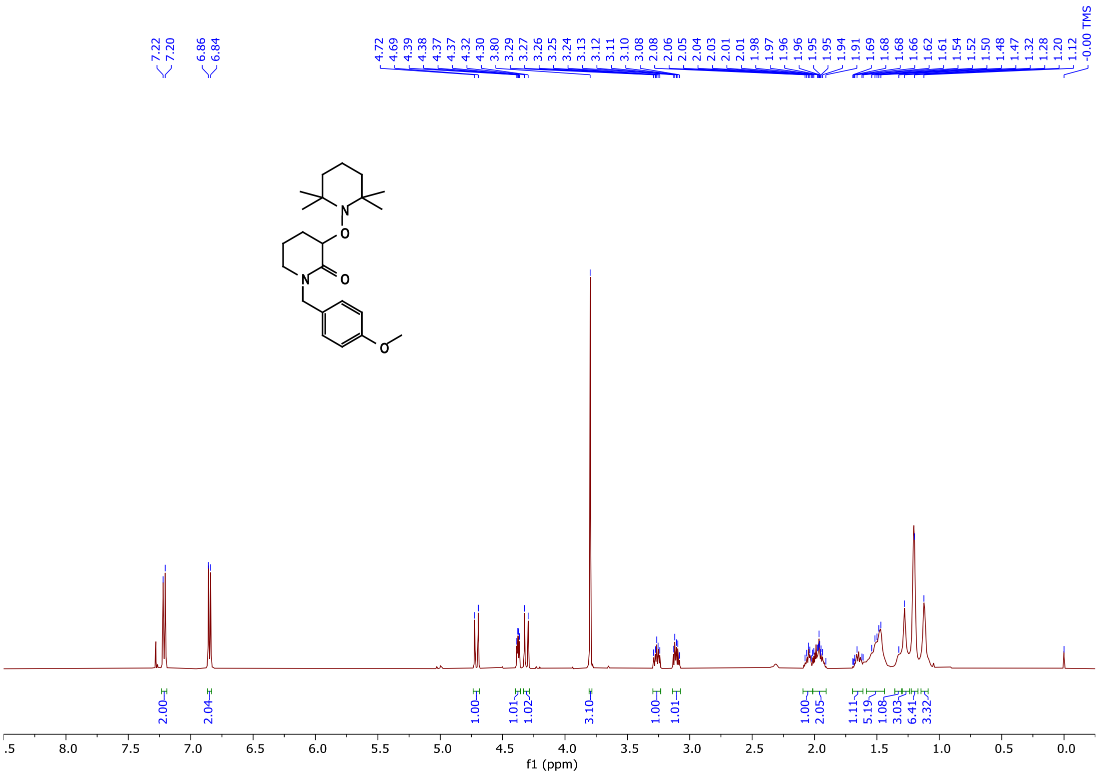

^{13}C{1H} NMR Spectrum of Compound 4d (126 MHz, $CDCl_3$)

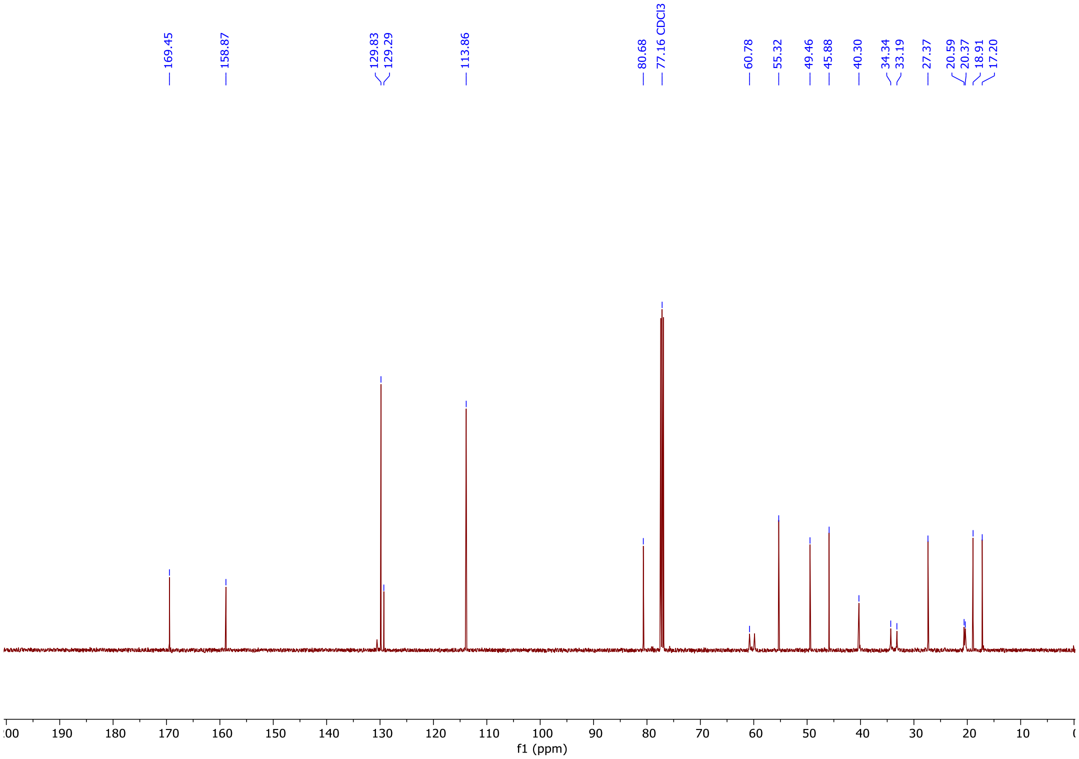

^{1}H NMR Spectrum of Compound 4e (500 MHz, $CDCl_3$)

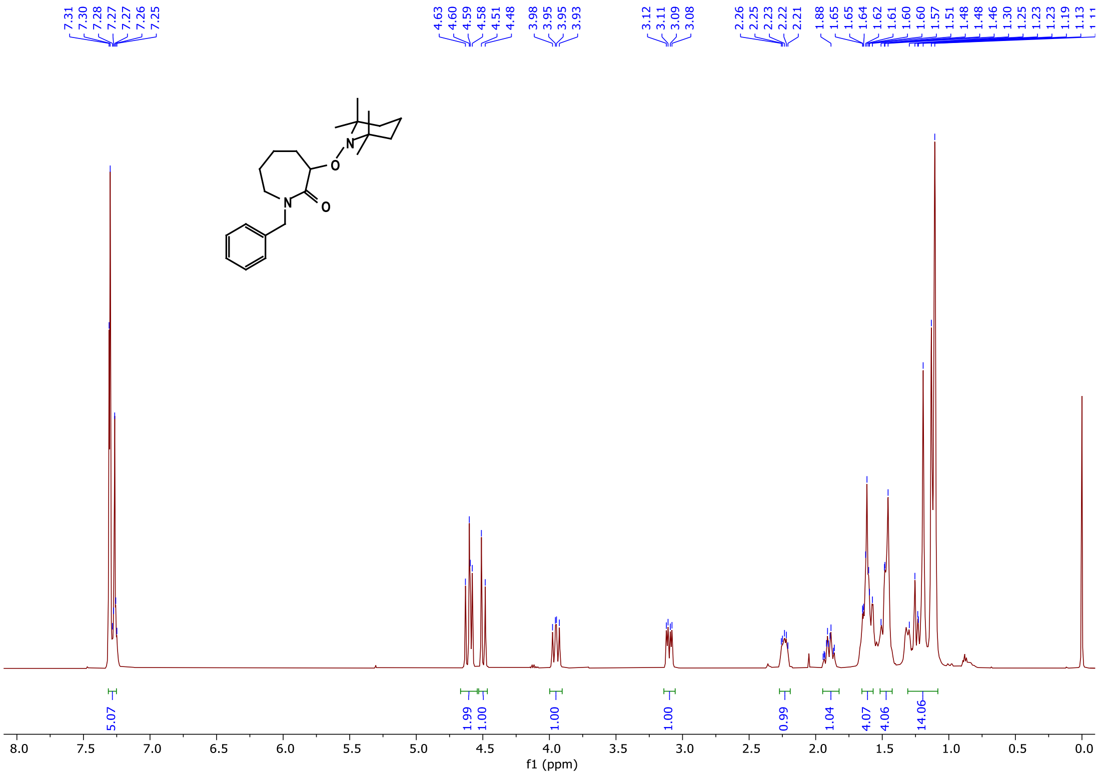

^{13}C{1H} NMR Spectrum of Compound 4e (126 MHz, $CDCl_3$)

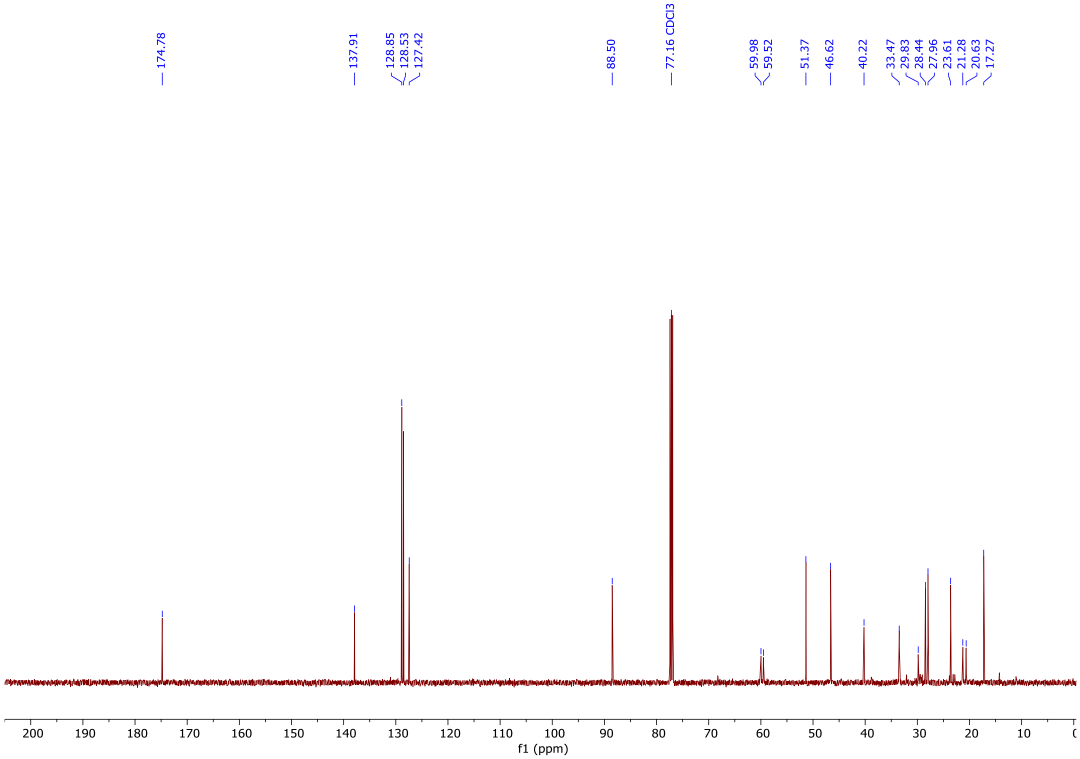

^{1}H NMR Spectrum of Compound *E-8* (500 MHz, CDCl₃)

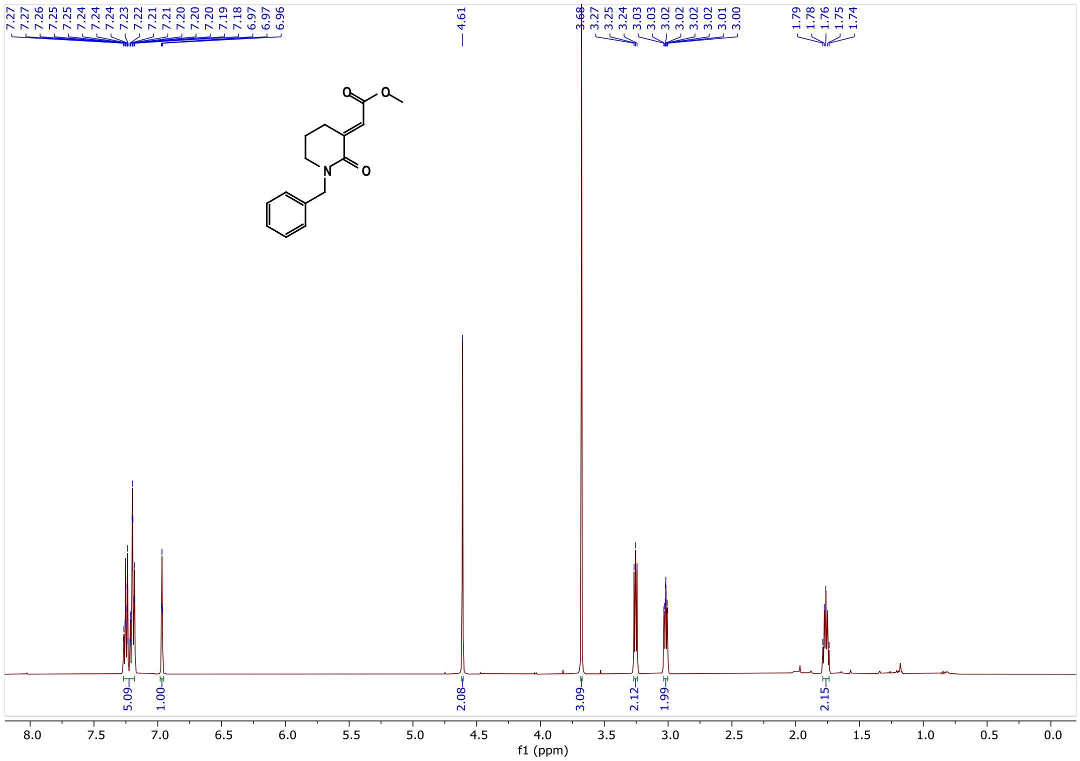

^{13}C{1H} NMR Spectrum of Compound *E-8* (126 MHz, CDCl₃)

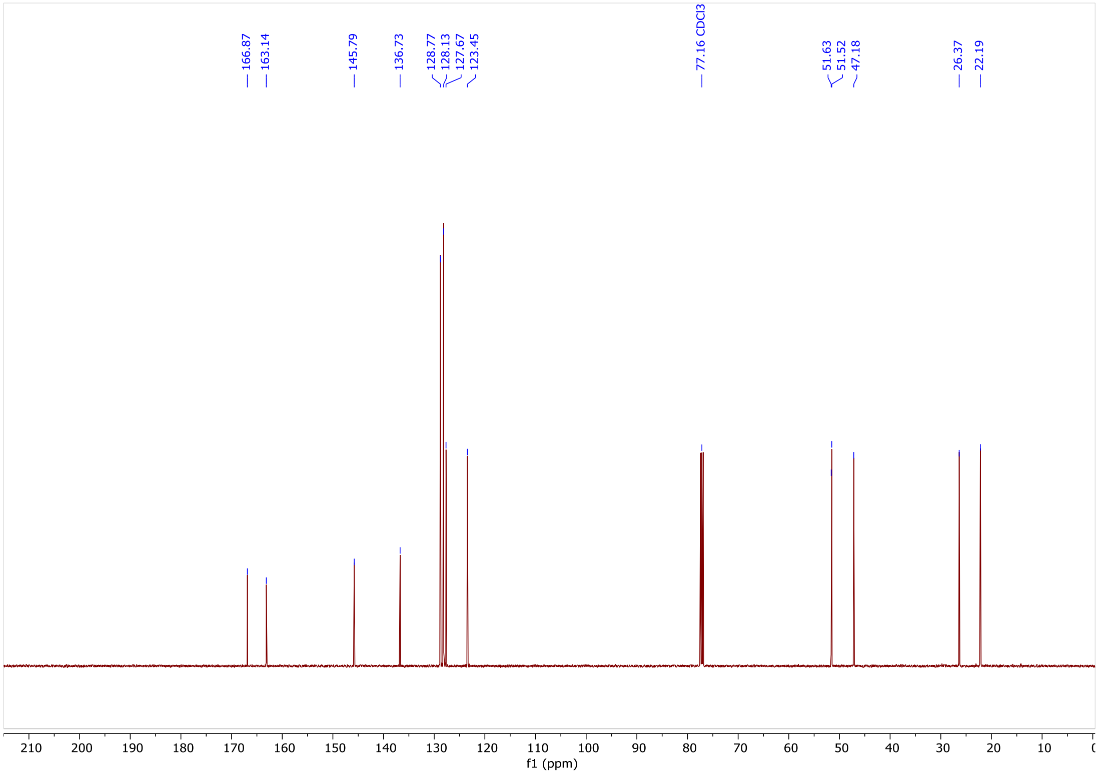

^{1}H NMR Spectrum of Compound *Z-8* (500 MHz, $CDCl_3$)

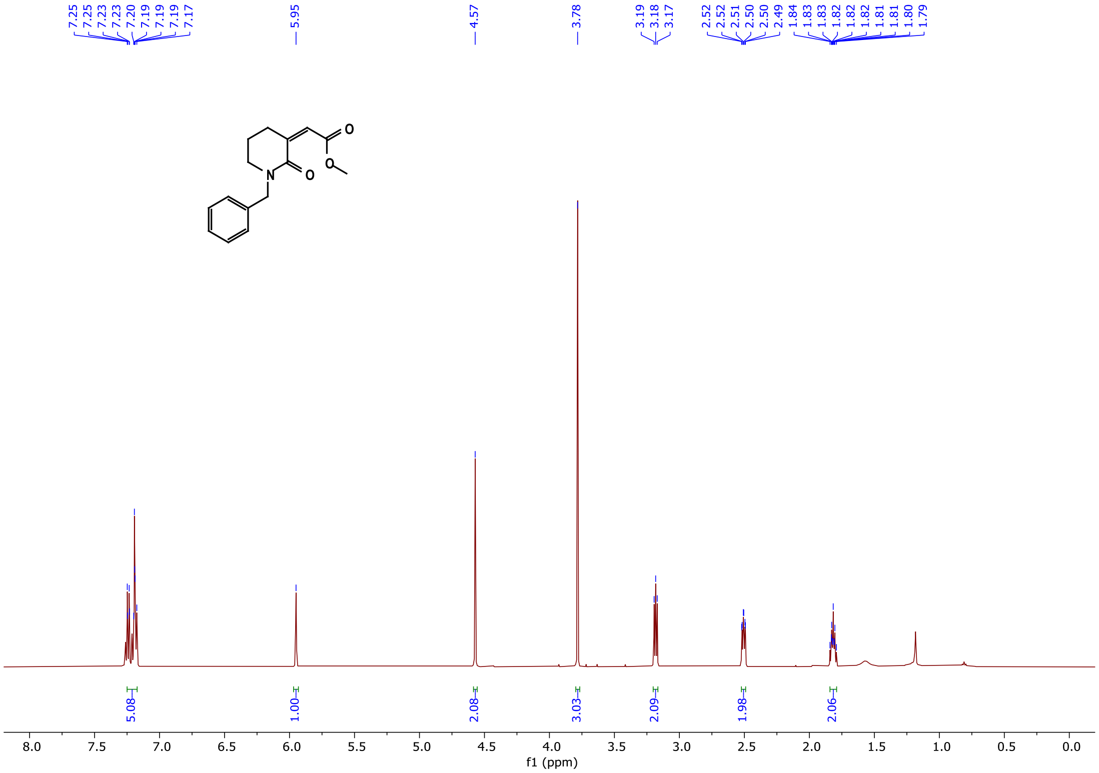

^{13}C{1H} NMR Spectrum of Compound *Z-8* (126 MHz, $CDCl_3$)

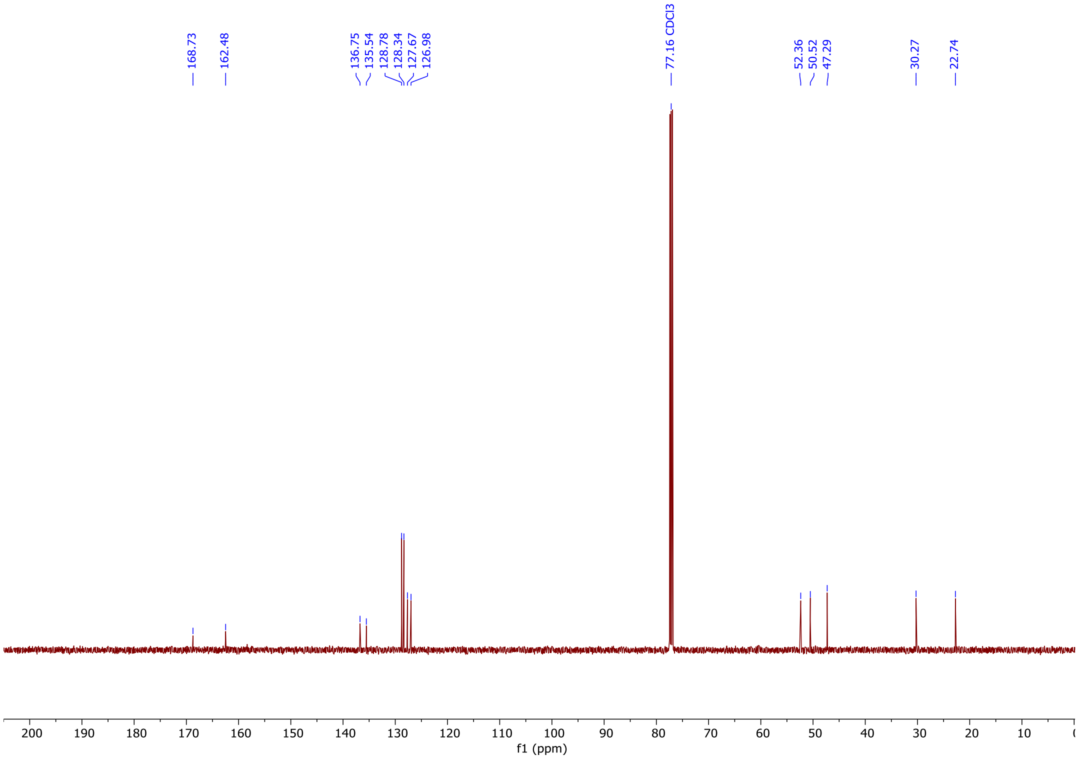

^{1}H NMR Spectrum of Compound 10 (500 MHz, $CDCl_3$)

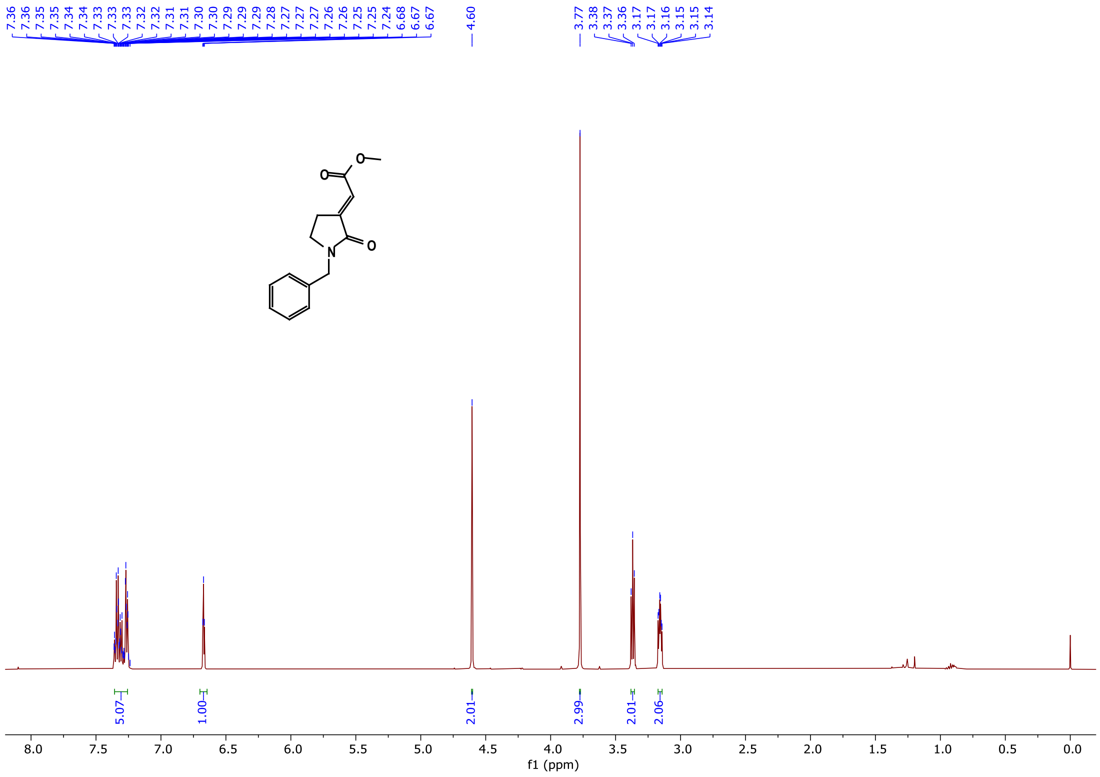

$^{13}C\{1H\}$ NMR Spectrum of Compound 10 (126 MHz, $CDCl_3$)

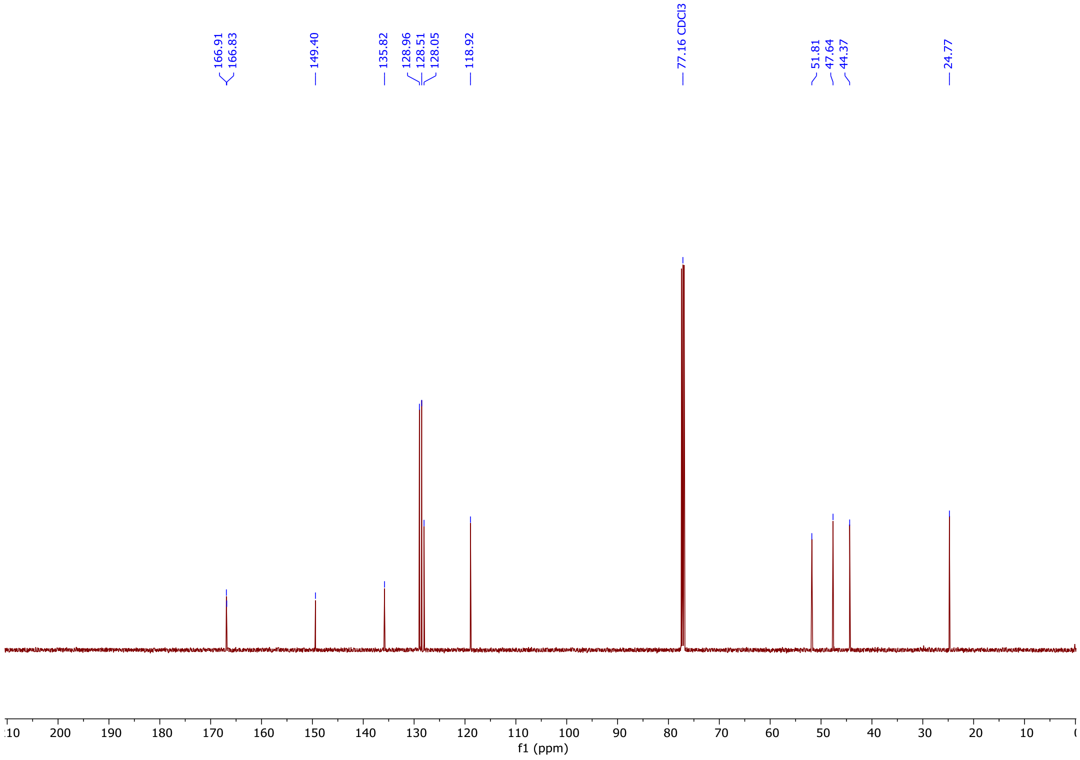

^{1}H NMR Spectrum of Compound 11 (500 MHz, $CDCl_3$)

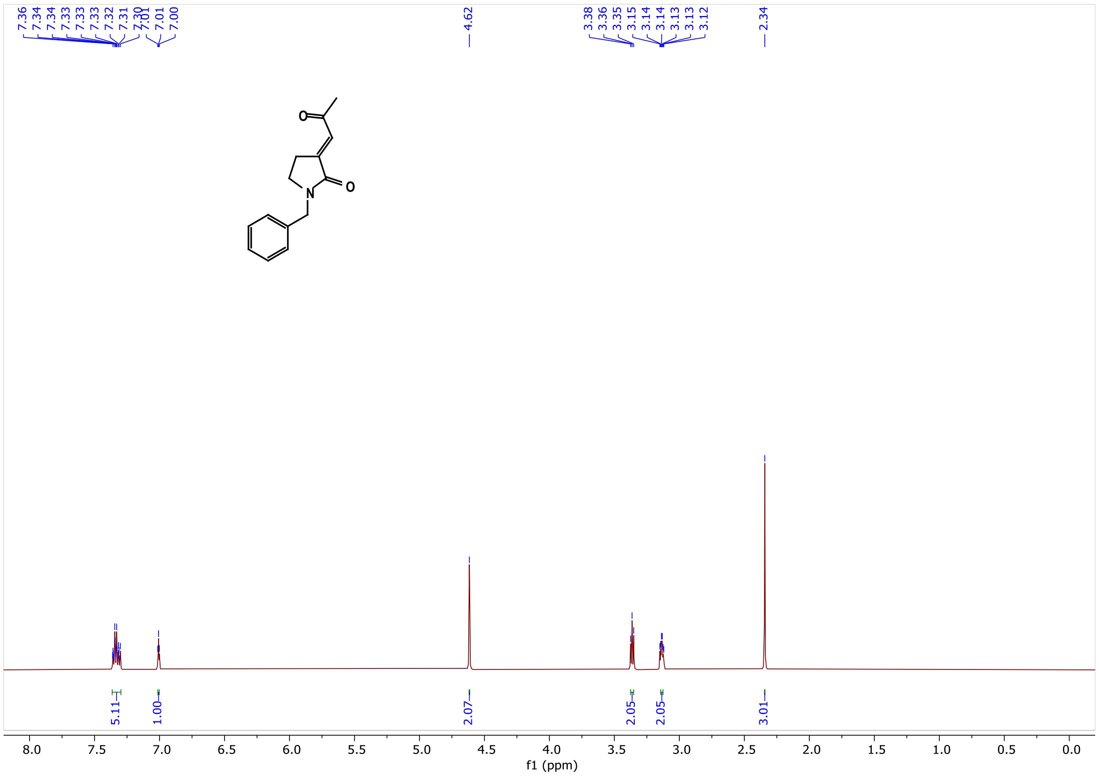

^{13}C{1H} NMR Spectrum of Compound 11 (126 MHz, $CDCl_3$)

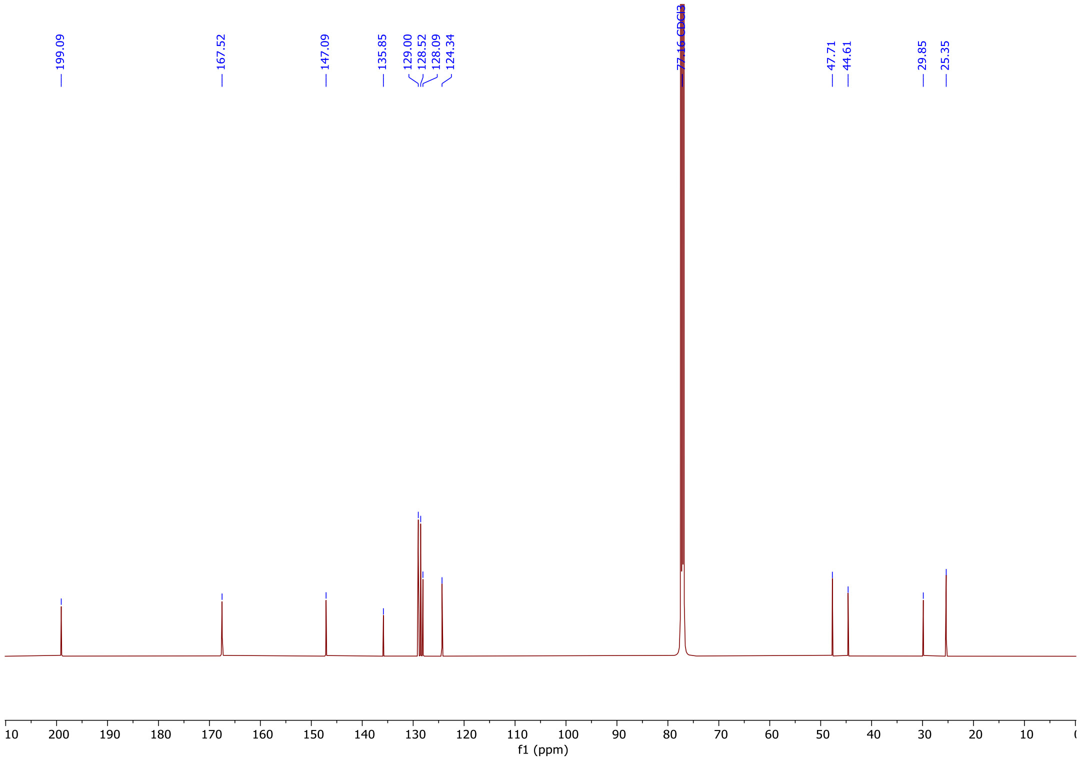

^{1}H NMR Spectrum of Compound 13 (500 MHz, $CDCl_3$)

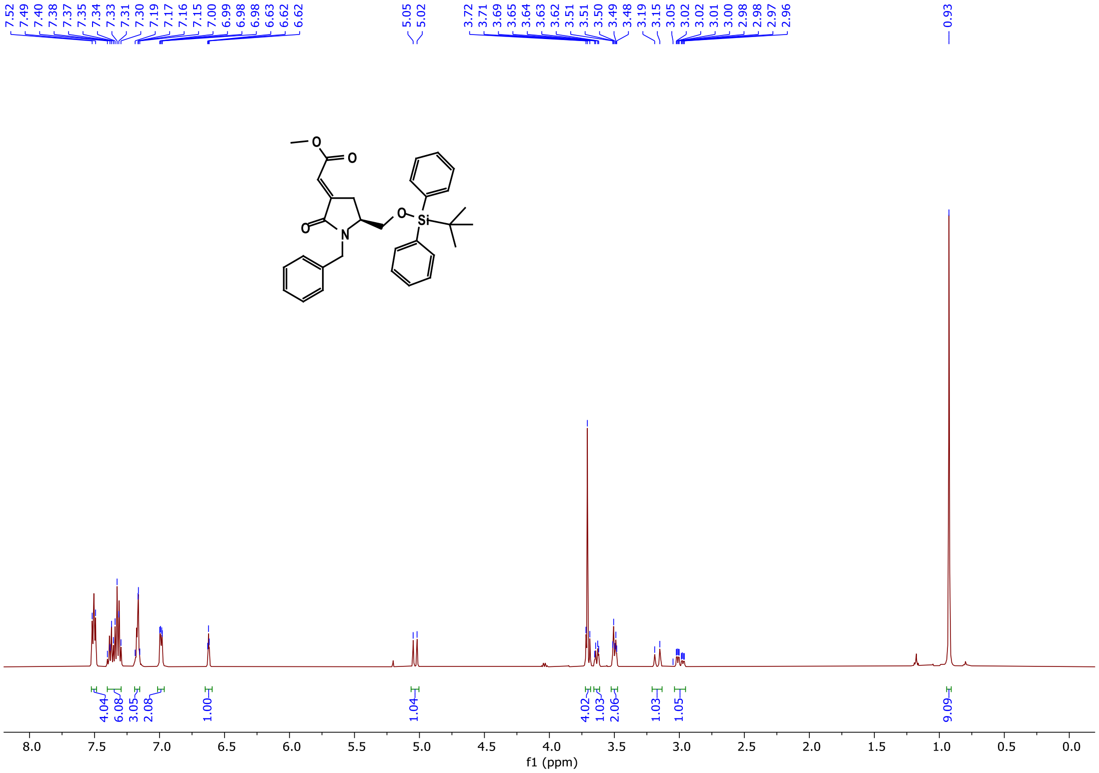

^{13}C{1H} NMR Spectrum of Compound 13 (126 MHz, $CDCl_3$)

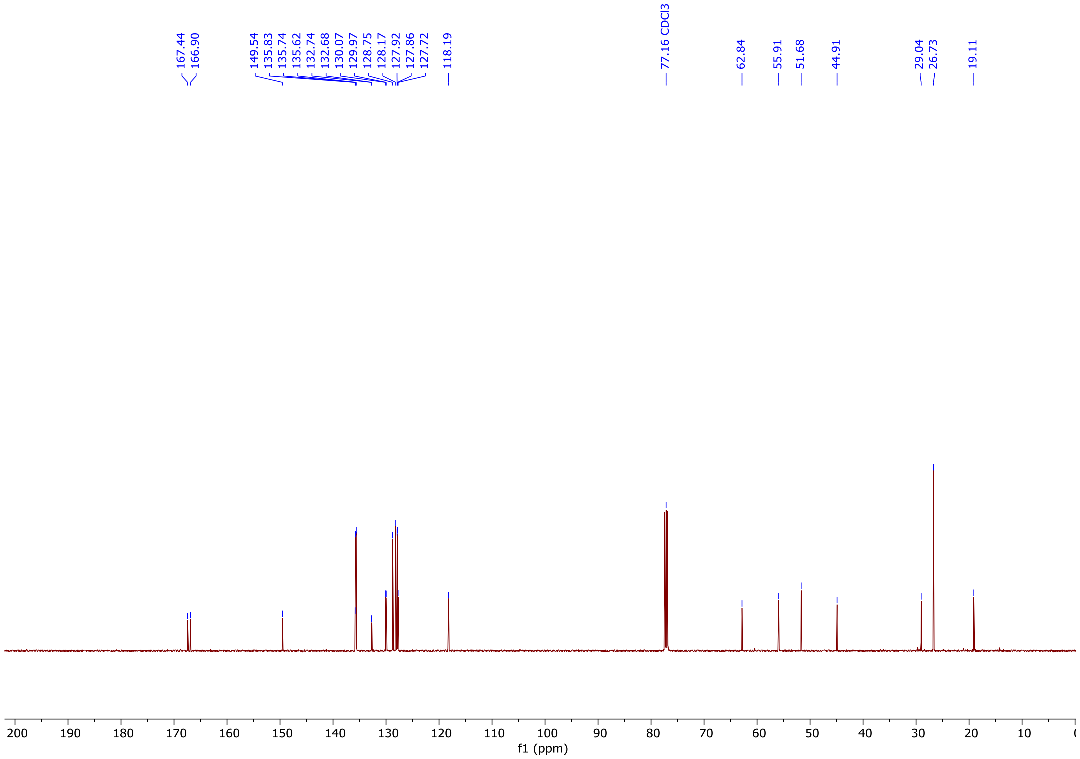

^{1}H NMR Spectrum of Compound 14 (500 MHz, $CDCl_3$)

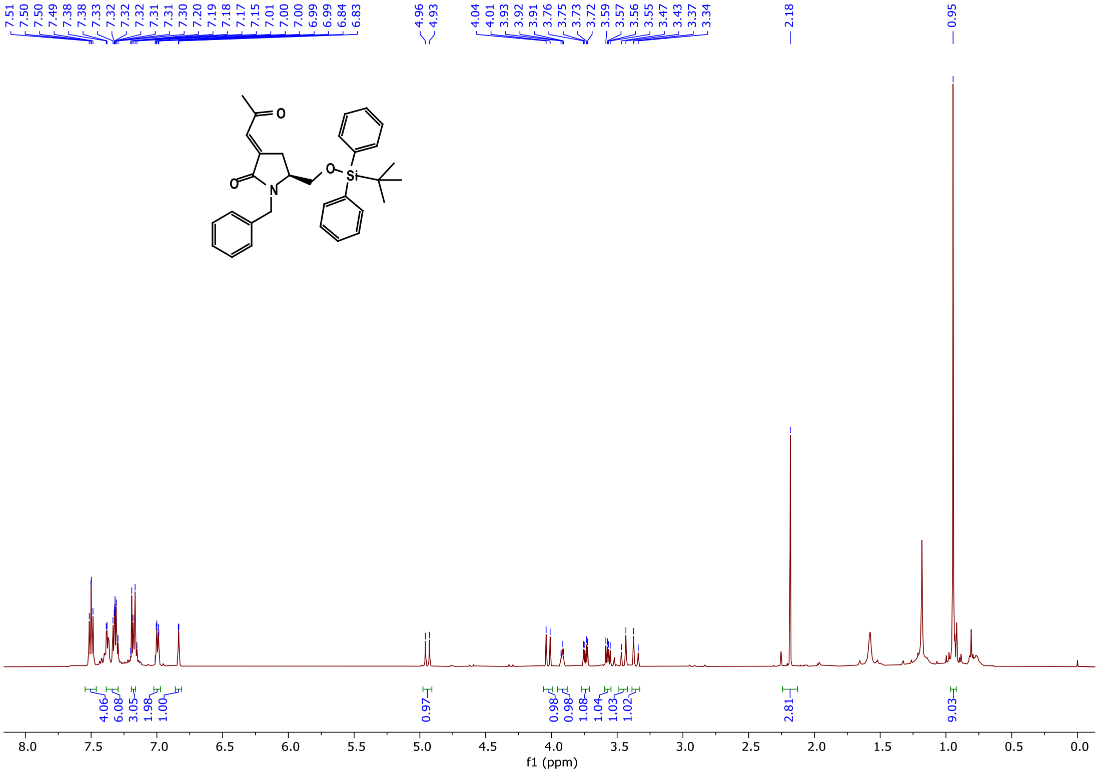

^{13}C{1H} NMR Spectrum of Compound 14 (126 MHz, $CDCl_3$)

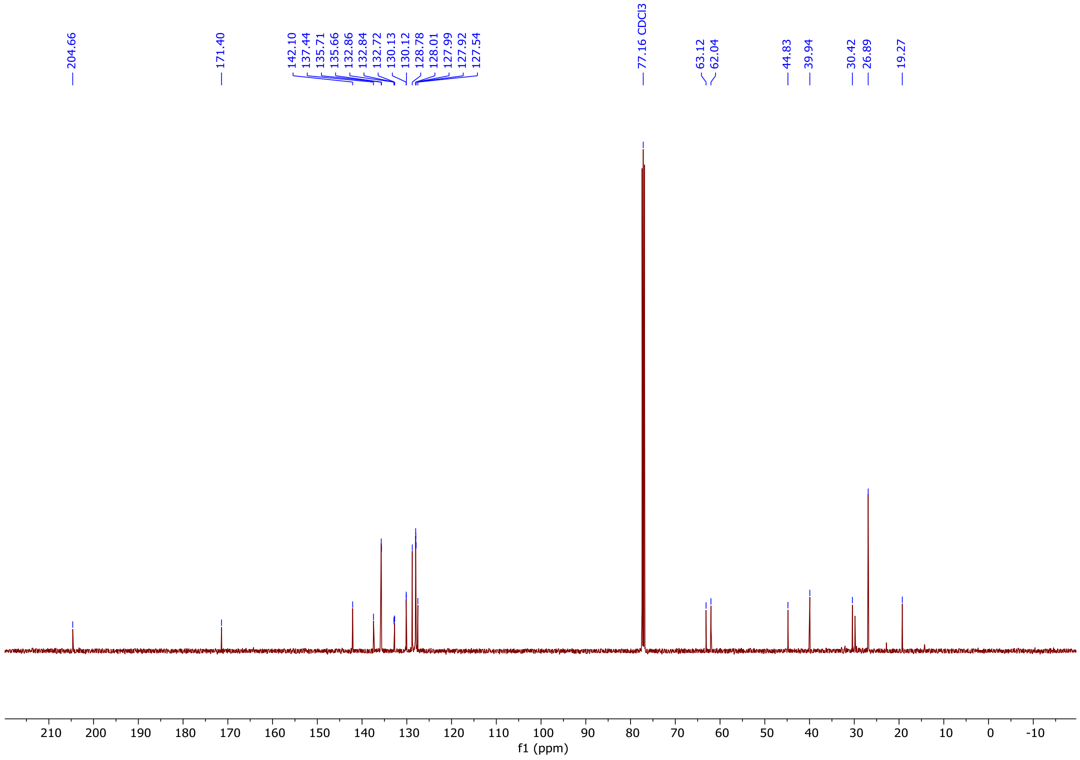

^{1}H NMR Spectrum of Compound 15 (500 MHz, $CDCl_3$)

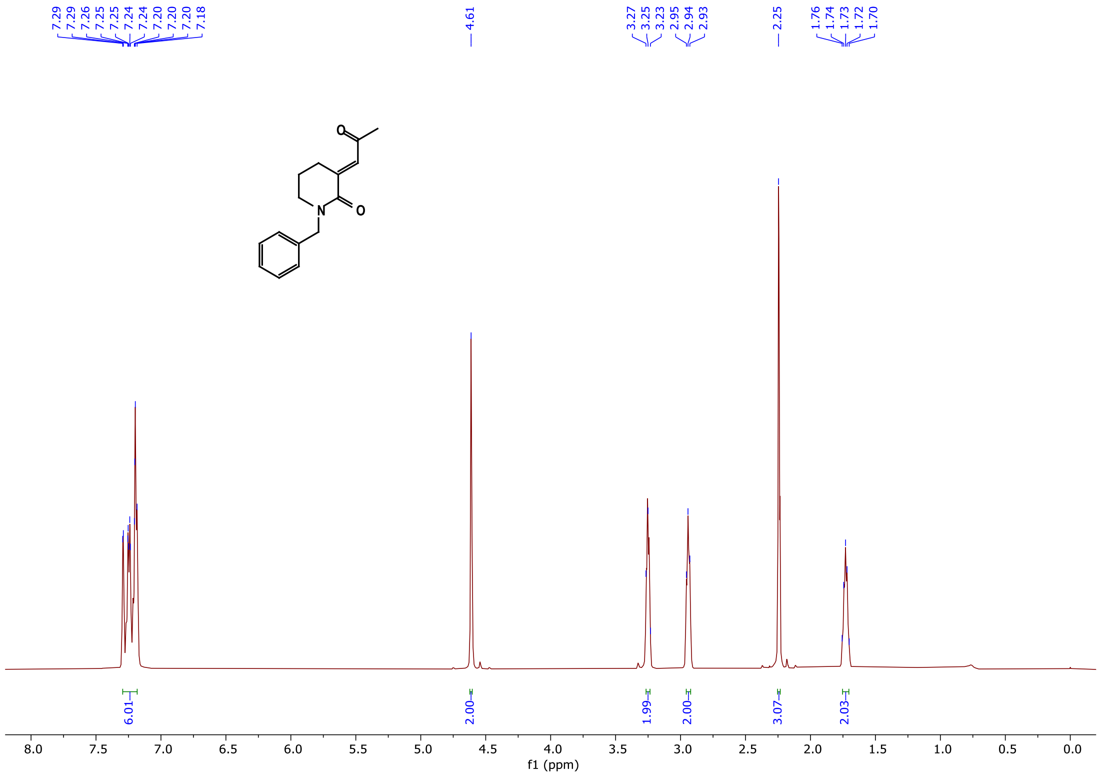

^{13}C{1H} NMR Spectrum of Compound 15 (126 MHz, $CDCl_3$)

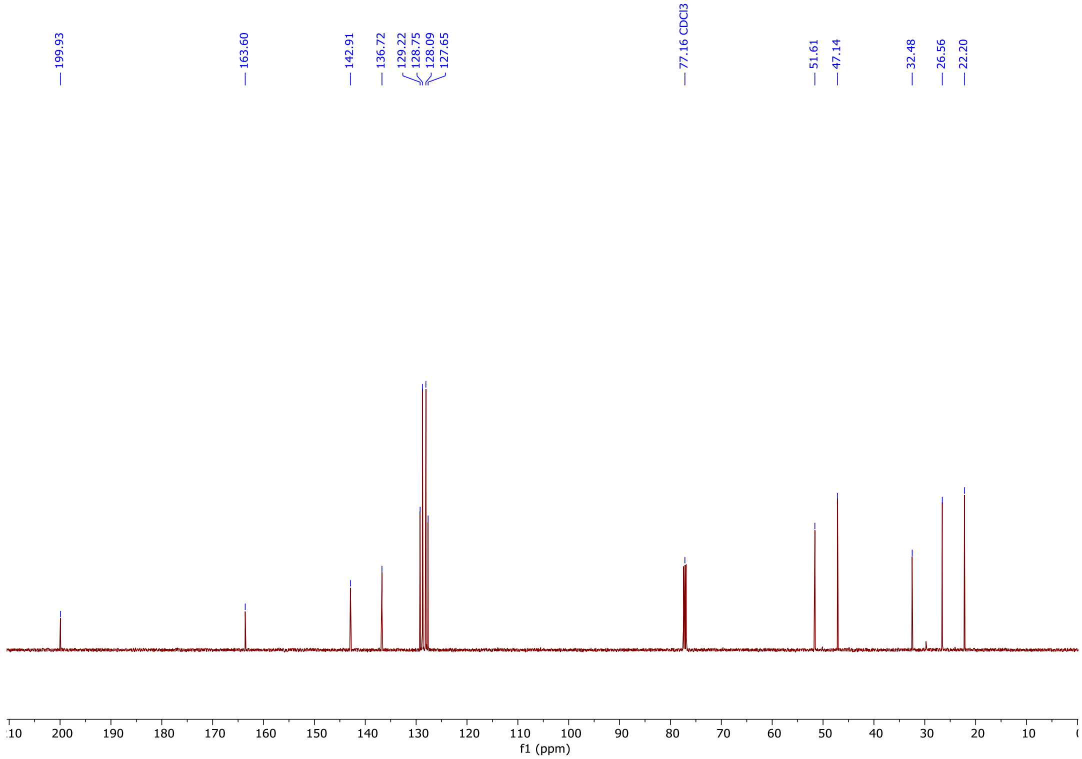

^{1}H NMR Spectrum of Compound 16 (500 MHz, $CDCl_3$)

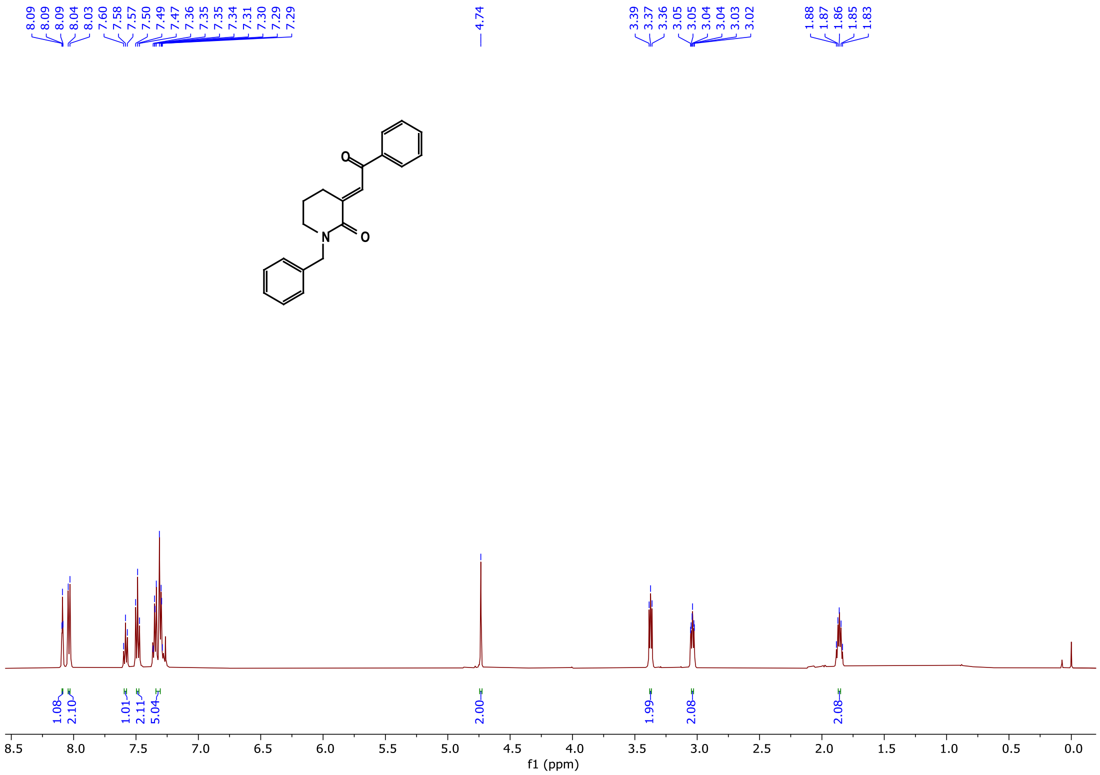

^{13}C{1H} NMR Spectrum of Compound 16 (126 MHz, $CDCl_3$)

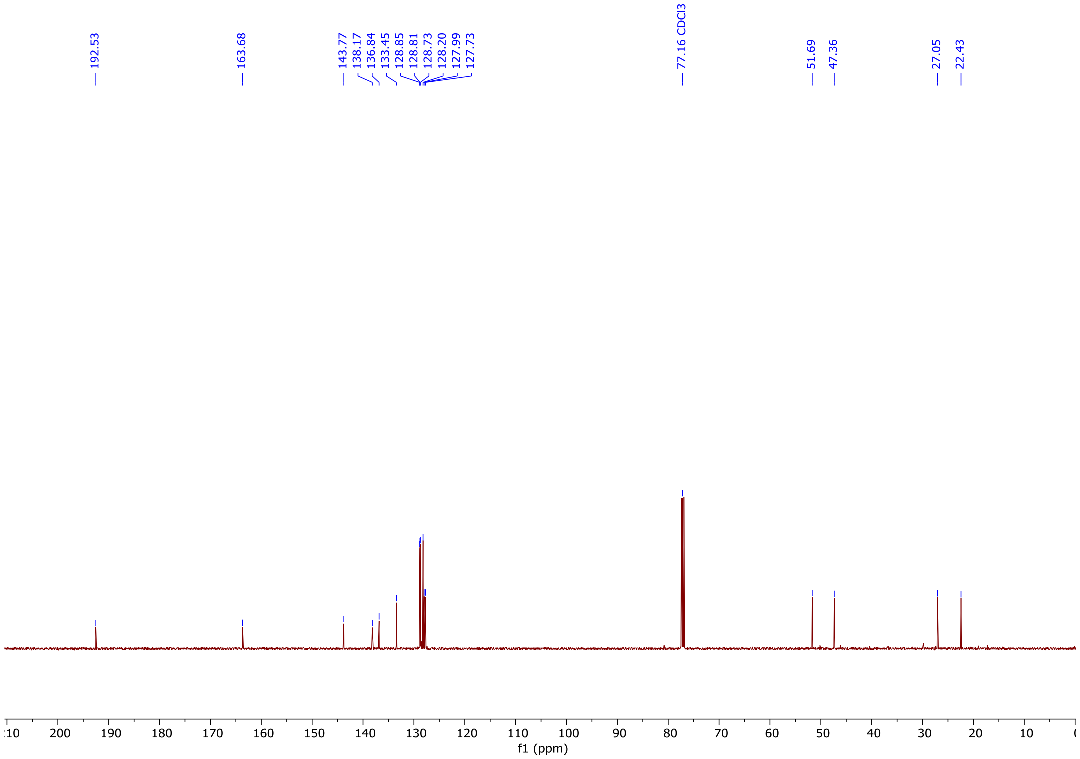

^{1}H NMR Spectrum of Compound 18 (500 MHz, $CDCl_3$)

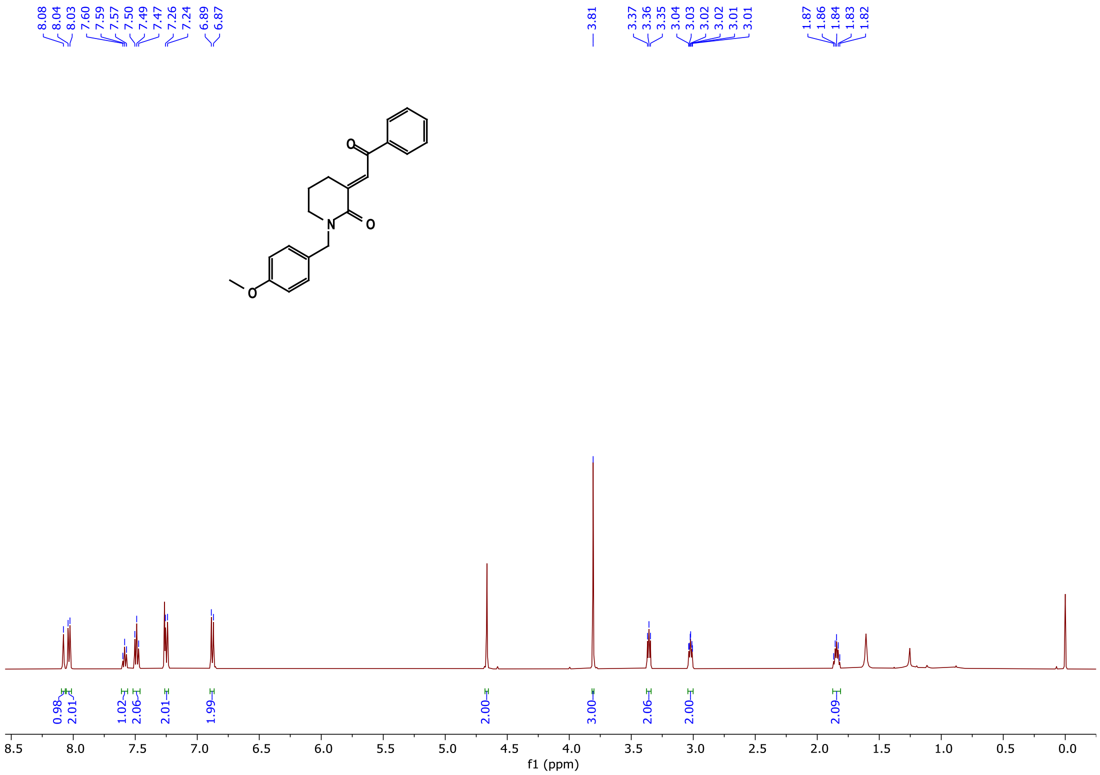

^{13}C{1H} NMR Spectrum of Compound 18 (126 MHz, $CDCl_3$)

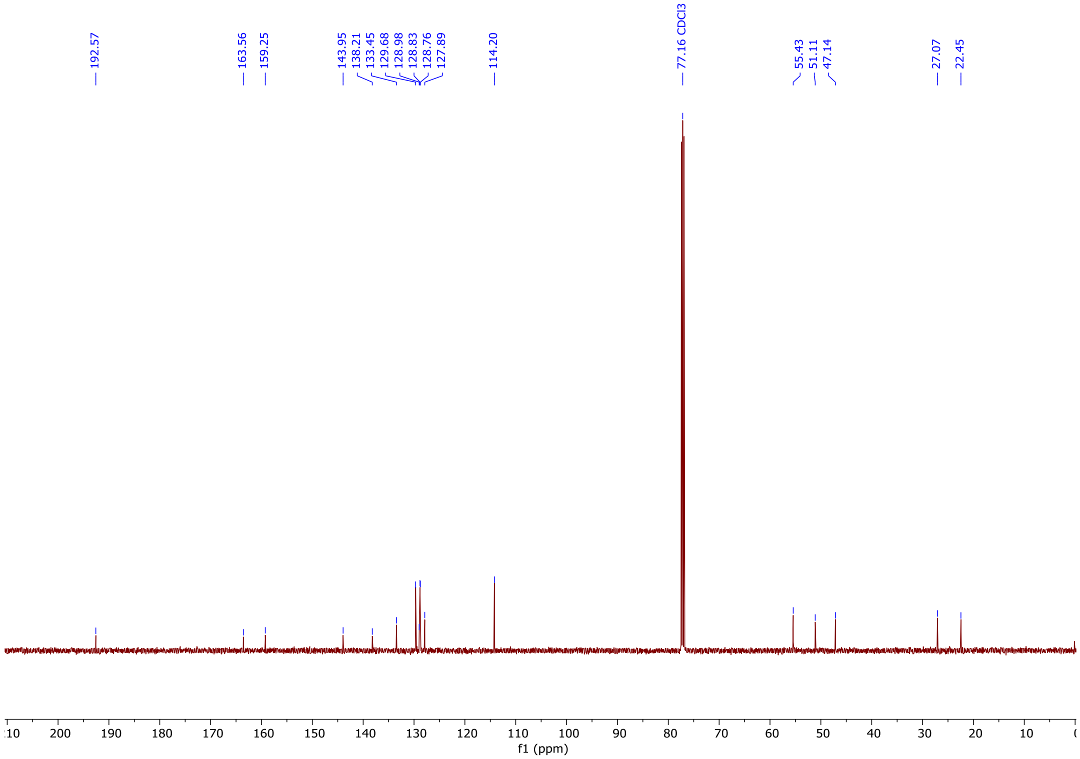

^{1}H NMR Spectrum of Compound 20 (500 MHz, $CDCl_3$)

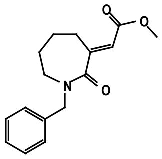

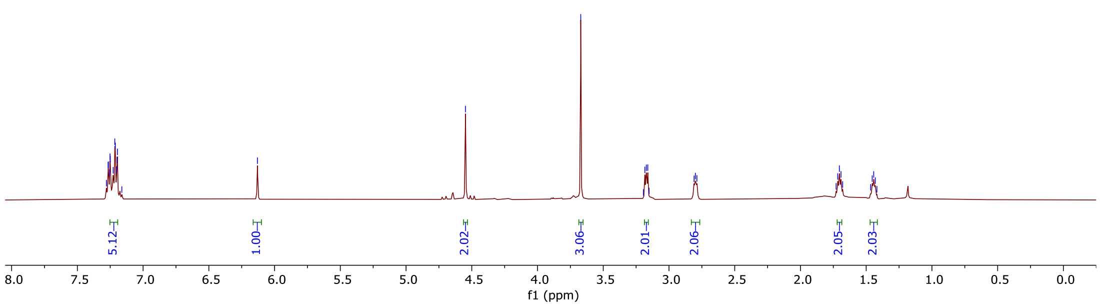

^{13}C{1H} NMR Spectrum of Compound 20 (126 MHz, $CDCl_3$)

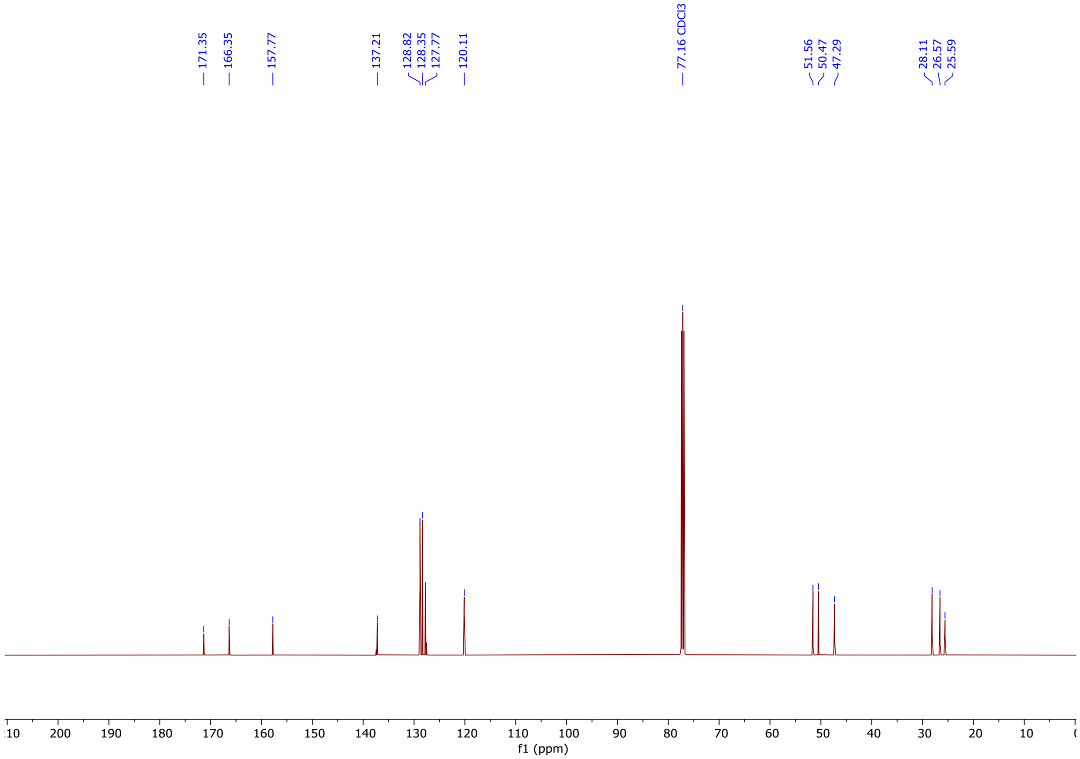

^{1}H NMR Spectrum of Compound 21 (500 MHz, $CDCl_3$)

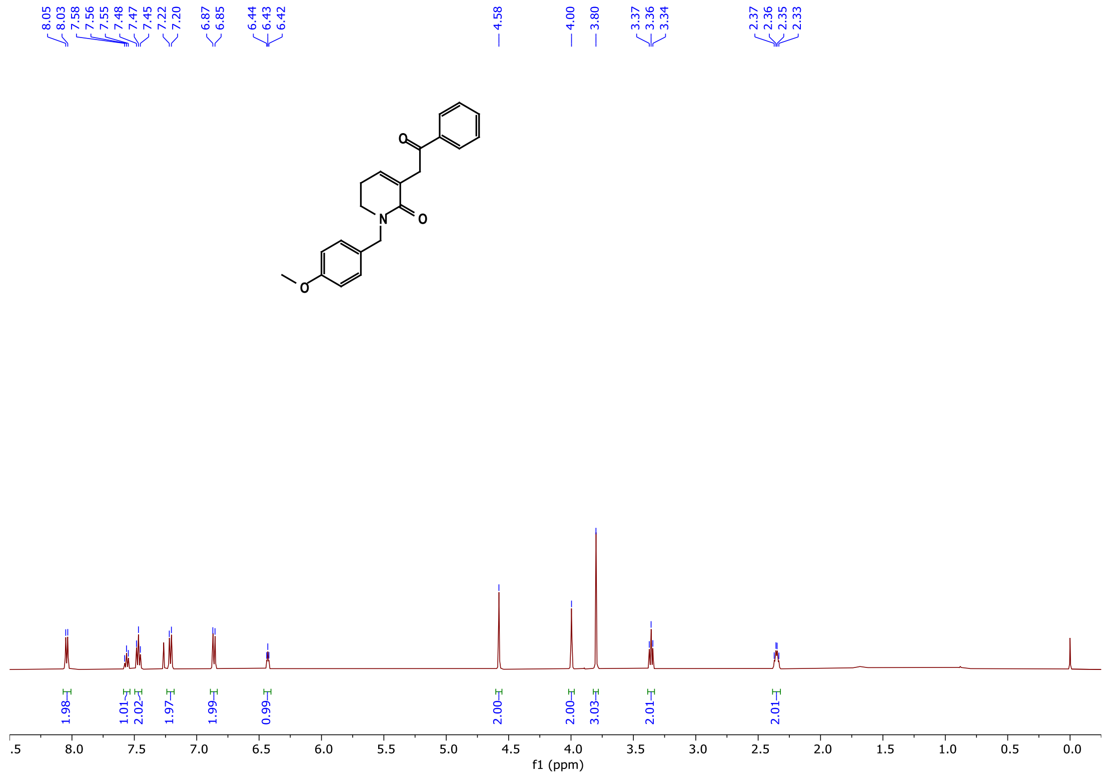

^{13}C{1H} NMR Spectrum of Compound 21 (126 MHz, $CDCl_3$)

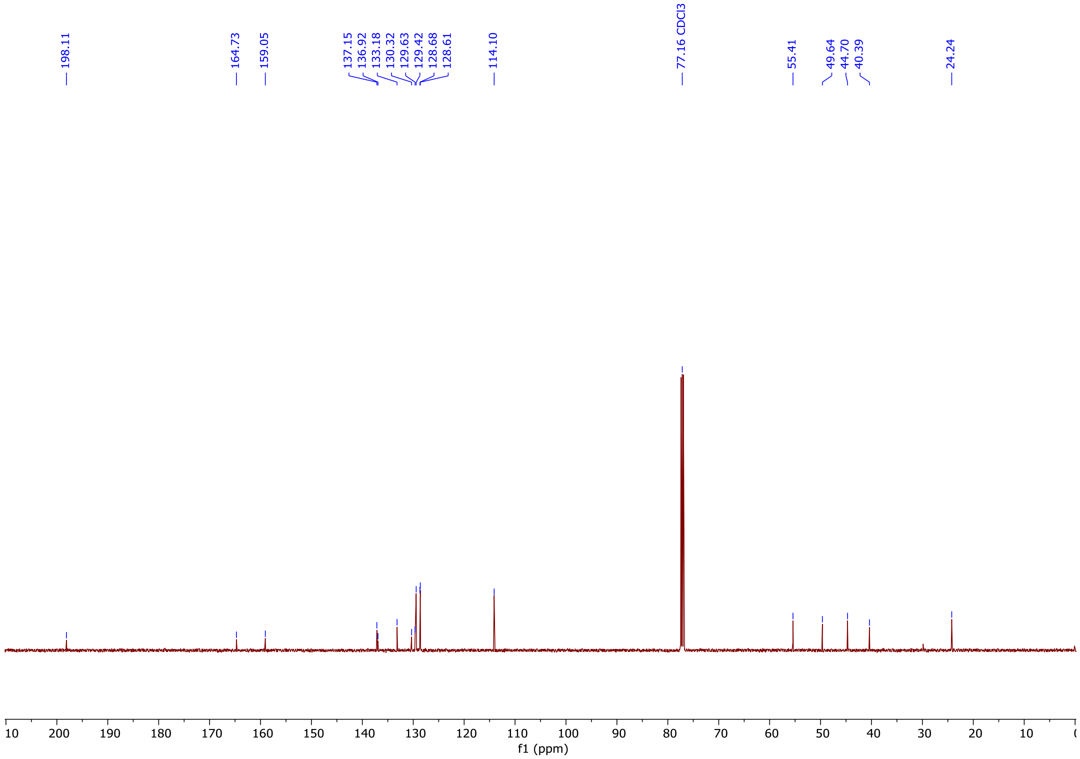

^{1}H NMR Spectrum of Callylactam A (500 MHz, $CDCl_3$)

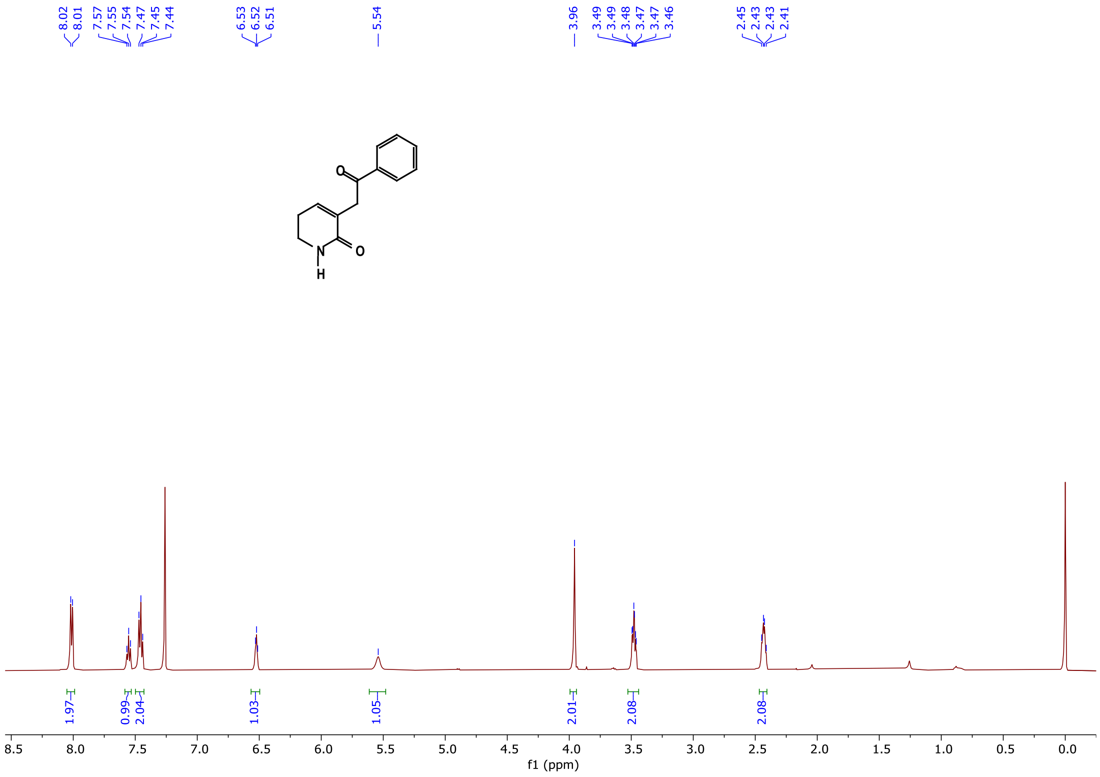

$^{13}C\{1H\}$ NMR Spectrum of Callylactam A (126 MHz, $CDCl_3$)

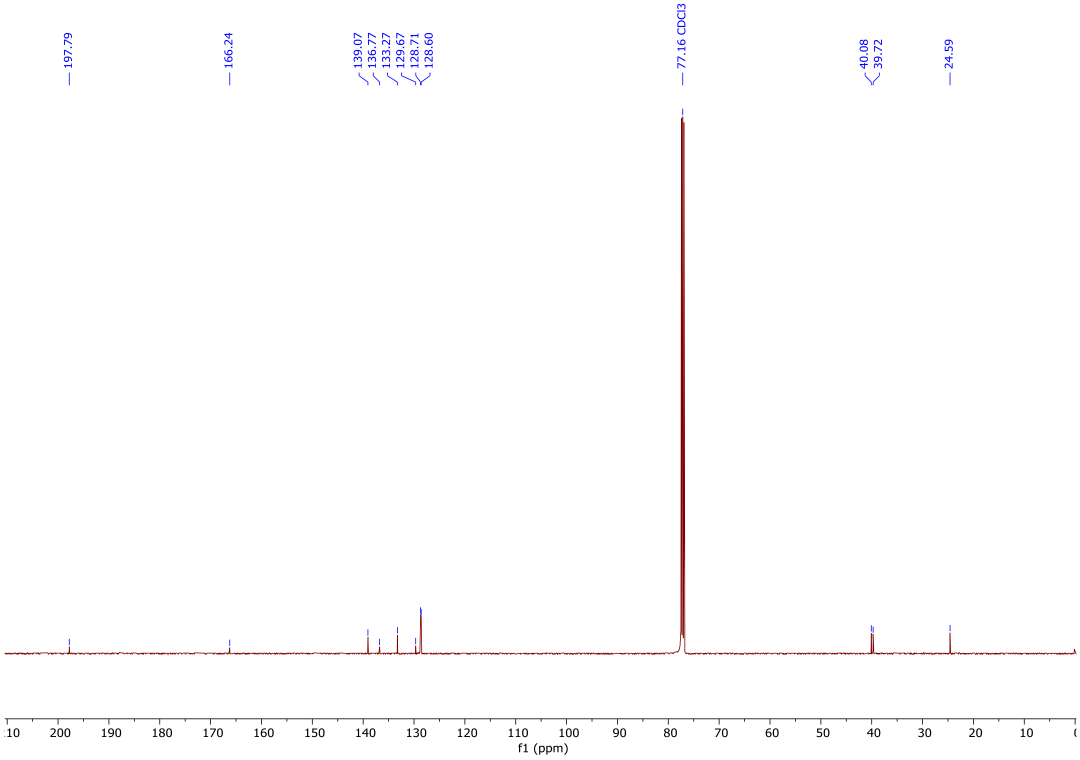

^{1}H NMR Spectrum of Callylactam A (500 MHz, CD_3OD)[4]

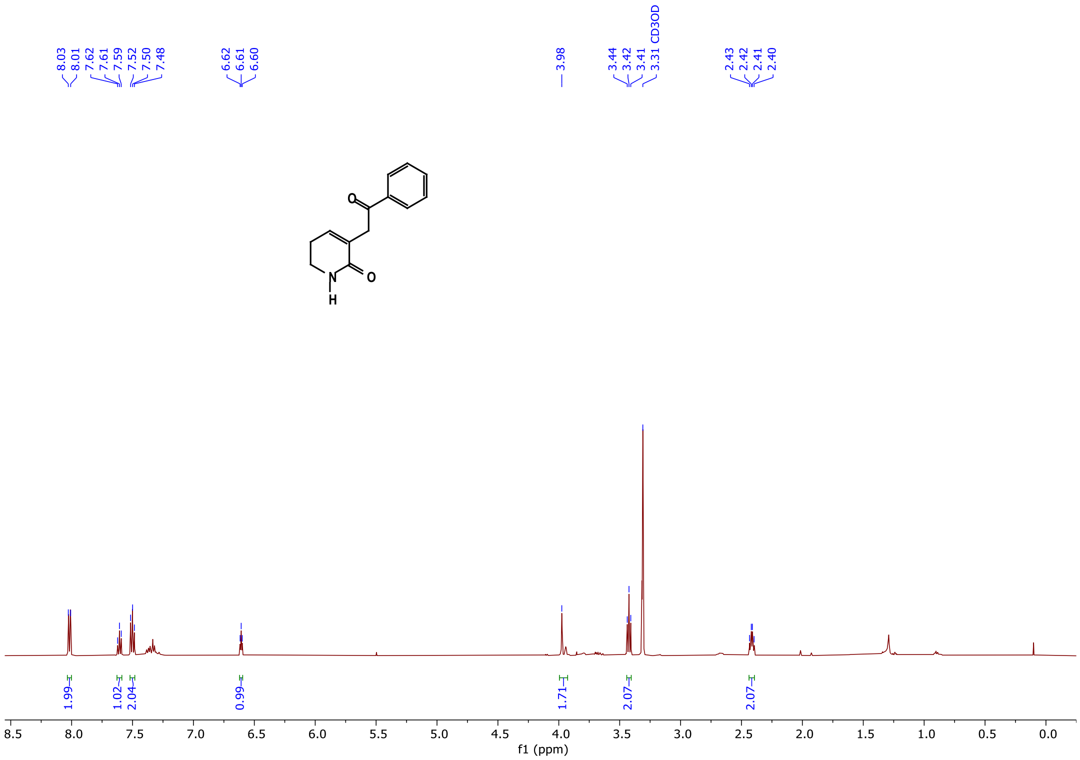

^{13}C{1H} NMR Spectrum of Callylactam A (126 MHz, CD_3OD)

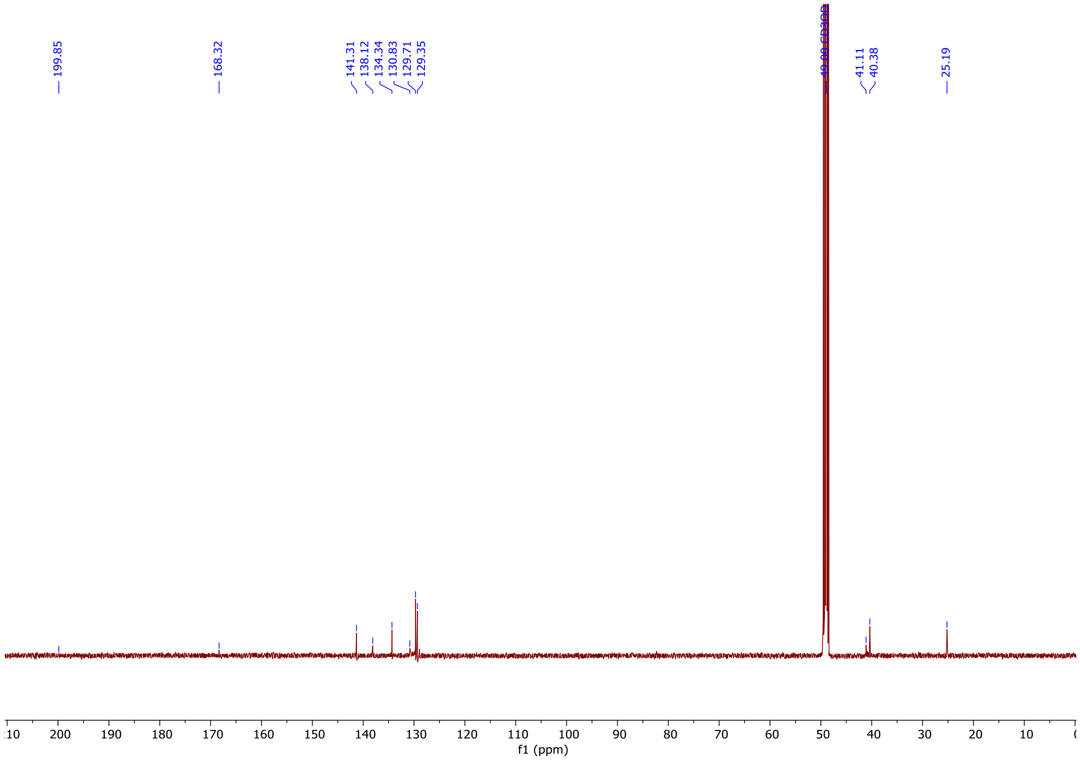

References

1. Volkov, A.; Buitrago, E.; Adolfsson, H. Direct Hydrosilylation of Tertiary Amides to Amines by an In Situ Formed Iron/N-Heterocyclic Carbene Catalyst. *Eur. J. Org. Chem.* **2013**, 2066.

2. Romero-Ibañez, J.; Cruz-Gregorio, S.; Quintero, L.; Sartillo-Piscil, F. Concise and Environmentally Friendly Asymmetric Total Synthesis of the Putative Structure of a Biologically Active 3-Hydroxy-2-piperidone Alkaloid. *Synthesis* **2018**, *50,* 2878.

3. Osorio-Nieto, U.; Chamorro-Arenas, D.; Quintero, L.; Höpfl, H.; Sartillo-Piscil, F. Transition Metal-Free Selective Double sp^3 C−H Oxidation of Cyclic Amines to 3-Alkoxyamine Lactams. *J. Org. Chem*. **2016**, *81*, 8625.

4. Yang, B.; Tao, H.; Zhou, X.; Lin, X.-P.; Liuy, Y. Two New Alkaloids from Marine Sponge *Callyspongia sp*. *Nat. Prod. Res*. **2013**, *27,* 433.
